# Predicted distribution and burden of podoconiosis in Cameroon

**DOI:** 10.1136/bmjgh-2018-000730

**Published:** 2018-06-22

**Authors:** Kebede Deribe, Jorge Cano, Abdel Jelil Njouendou, Mathias Esum Eyong, Amuam Andrew Beng, Emanuele Giorgi, David M Pigott, Rachel L Pullan, Abdisalan M Noor, Fikre Enquselassie, Christopher J L Murray, Simon I Hay, Melanie J Newport, Gail Davey, Samuel Wanji

**Affiliations:** 1 Wellcome Trust Brighton and Sussex Centre for Global Health Research, Brighton and Sussex Medical School, Brighton, UK; 2 School of Public Health, Addis Ababa University, Addis Ababa, Ethiopia; 3 Department of Disease Control, London School of Hygiene and Tropical Medicine, London, UK; 4 Parasites and Vector Biology Research Unit (PAVBRU), Department of Microbiology and Parasitology, University of Buea, Buea, Cameroon; 5 Research Foundation for Tropical Diseases and the Environment (REFOTDE), Buea, Cameroon; 6 Lancaster Medical School, Faculty of Health and Medicine, Lancaster University, Lancaster, UK; 7 Institute for Health Metrics and Evaluation, University of Washington, Seattle, Washington, USA; 8 Kenya Medical Research Institute–Wellcome Trust Collaborative Programme, Nairobi, Kenya; 9 Centre for Tropical Medicine and Global Health, Nuffield Department of Clinical Medicine, University of Oxford, Oxford, UK; 10 Big Data Institute, Li Ka Shing Centre for Health Information and Discovery, University of Oxford, Oxford, UK

**Keywords:** podoconiosis, non-filarial, elephantiasis, lymphedema, cameroon, mapping

## Abstract

**Introduction:**

Understanding the number of cases of podoconiosis, its geographical distribution and the population at risk are crucial to estimating the burden of this disease in endemic countries. We assessed each of these using nationwide data on podoconiosis prevalence in Cameroon.

**Methods:**

We analysed data arising from two cross-sectional surveys in Cameroon. The dataset was combined with a suite of environmental and climate data and analysed within a robust statistical framework, which included machine learning-based approaches and geostatistical modelling. The environmental limits, spatial variation of predicted prevalence, population at risk and number of cases of podoconiosis were each estimated.

**Results:**

A total of 214 729 records of individuals screened for podoconiosis were gathered from 748 communities in all 10 regions of Cameroon. Of these screened individuals, 882 (0.41%; 95% CI 0.38 to 0.44) were living with podoconiosis. High environmental suitability for podoconiosis was predicted in three regions of Cameroon (Adamawa, North West and North). The national population living in areas environmentally suitable for podoconiosis was estimated at 5.2 (95% CI 4.7 to 5.8) million, which corresponds to 22.3% of Cameroon’s population in 2015. Countrywide, in 2015, the number of adults estimated to be suffering from podoconiosis was 41 556 (95% CI, 1170 to 240 993). Four regions (Central, Littoral, North and North West) contributed 61.2% of the cases.

**Conclusion:**

In Cameroon, podoconiosis is more widely distributed geographically than was initially expected. The number of cases and the population at risk are considerable. Expanding morbidity management and follow-up of cases is of utmost necessity. Promotion of footwear use and regular foot hygiene should be at the forefront of any intervention plan.

**Key questions**

**What is already known?**

- Previous studies have documented that, although prevalence is low, podoconiosis is widespread in Cameroon.
- This is the first comprehensive analysis using modelling and nationwide data to estimate the population at risk and burden of podoconiosis in Cameroon.

**What are the new findings?**

- Our model prediction suggests marked ecological limits and identified environmental drivers of podoconiosis in Cameroon.
- In 2015 in Cameroon, there were 5.2 (95% CI 4.7 to 5.8) million people at risk and 41 556 (95% CI, 1170 to 240 993) podoconiosis cases.

**What do the new findings imply?**

- The findings presented here indicate the need to scale up interventions including early diagnosis, morbidity management and follow-up of cases.

## Introduction

Neglected tropical diseases affect more than 1 billion people, the vast majority of whom are among the poorest living within endemic countries.^1^ The neglected tropical disease podoconiosis is one of the principal causes of tropical lymphoedema,^2 3^ which can lead to massive swelling of the lower legs with subsequent suffering to those affected.^2 4^ The disease is found in highland areas of tropical Africa, Central America and limited areas of India (north-west) and south-eastern Asia, according to WHO.^5 6^ However, the actual geographical distribution and burden remain unknown in most endemic areas. Determining the burden and geographical distribution of podoconiosis is of utmost importance to guide resource allocation and to monitor and evaluate the impact of prevention and control interventions.^2 5^ Additionally, estimating the number of potential cases has shown to help strengthen active surveillance and inform national control strategies and case enrolment.^7 8^

Podoconiosis is one of the diseases with potential for elimination.^9^ It can easily be prevented through the consistent use of footwear starting in early childhood, coupled with proper foot hygiene. Those who have developed the disease can reduce their likelihood of morbidity and disability through hygiene-based management.^2^ This includes foot hygiene, custom-made footwear, bandaging, exercise and elevation, wound care and prevention and management of acute attacks. Previous studies have documented the success of these strategies in improving quality of life and reduction of morbidity burden.^9–11^

Podoconiosis is caused by long-term exposure to red clay soils, with mineral particle-induced inflammation on a background of genetic susceptibility.^12–17^ Interactions between genetic and environmental factors trigger an inflammatory response that leads to lymphoedema and fibrosis.^2^ It is hypothesised that mineral particles that penetrate bare skin are engulfed by macrophages in the lower limb lymphatics and induce an inflammatory response in the lymphatic vessels. This is followed by fibrosis and obstruction of the vessel lumen leading to oedema of the lower leg, which progresses into elephantiasis.^2^

Certain types of soils such as clay and silt have proven to be associated with a higher risk of podoconiosis.^13–15^ Thus, soils that are fine textured and sticky in nature are more easily able to penetrate the skin and become absorbed within the body.^18^ Rainfall, altitude, terrain slope and some types of land cover have been found to favour the occurrence of podoconiosis.^13–15^ All these factors ultimately contribute to the type of soils generated.^14^

Nowadays, the availability of geographical data on soil composition, climate (ie, temperature and precipitation) and topography, primarily derived from remotely sensed data, and the development of robust statistical and modelling approaches are making the study of the relative contribution of all these environmental factors possible.^7 14 15^ Studies conducted in Ethiopia, a country that is thought to bear the highest burden of podoconiosis, have enabled identification of up to eight environmental factors (elevation and derived slope, annual precipitation, Enhanced Vegetation Index (EVI), clay and silt content of the top soil, population density and distance from water bodies) driving the distribution of podoconiosis across the country.^14^ Other research carried out in Ethiopia, but on a more local scale, showed that soil chemicals such as smectite quartz and mica, present in clay-rich soils, were strongly associated with the occurrence of podoconiosis.^15^ However, it is likely that these factors and others that may not have been reported yet do not equally influence the distribution of podoconiosis everywhere. Therefore, identifying environmental factors that determine the distribution of podoconiosis in distinctive geographic areas should be considered a prerequisite for delineating the global distribution of podoconiosis.^2 5 14^

In Cameroon, another podoconiosis-endemic country, a few studies have been conducted in the north-west of the country.^19–21^ Yet, the presence of podoconiosis elsewhere in Cameroon and the environmental drivers underpinning its distribution remain to be determined. We use machine learning and geostatistical methods^7 13 14^ and podoconiosis prevalence data collected in two surveys in Cameroon to (1) identify the environmental drivers of podoconiosis, (2) determine its geographical limits and finally (3) estimate the disease burden in environmentally suitable areas.

## Methods

### Podoconiosis prevalence data

**Figure 1:**
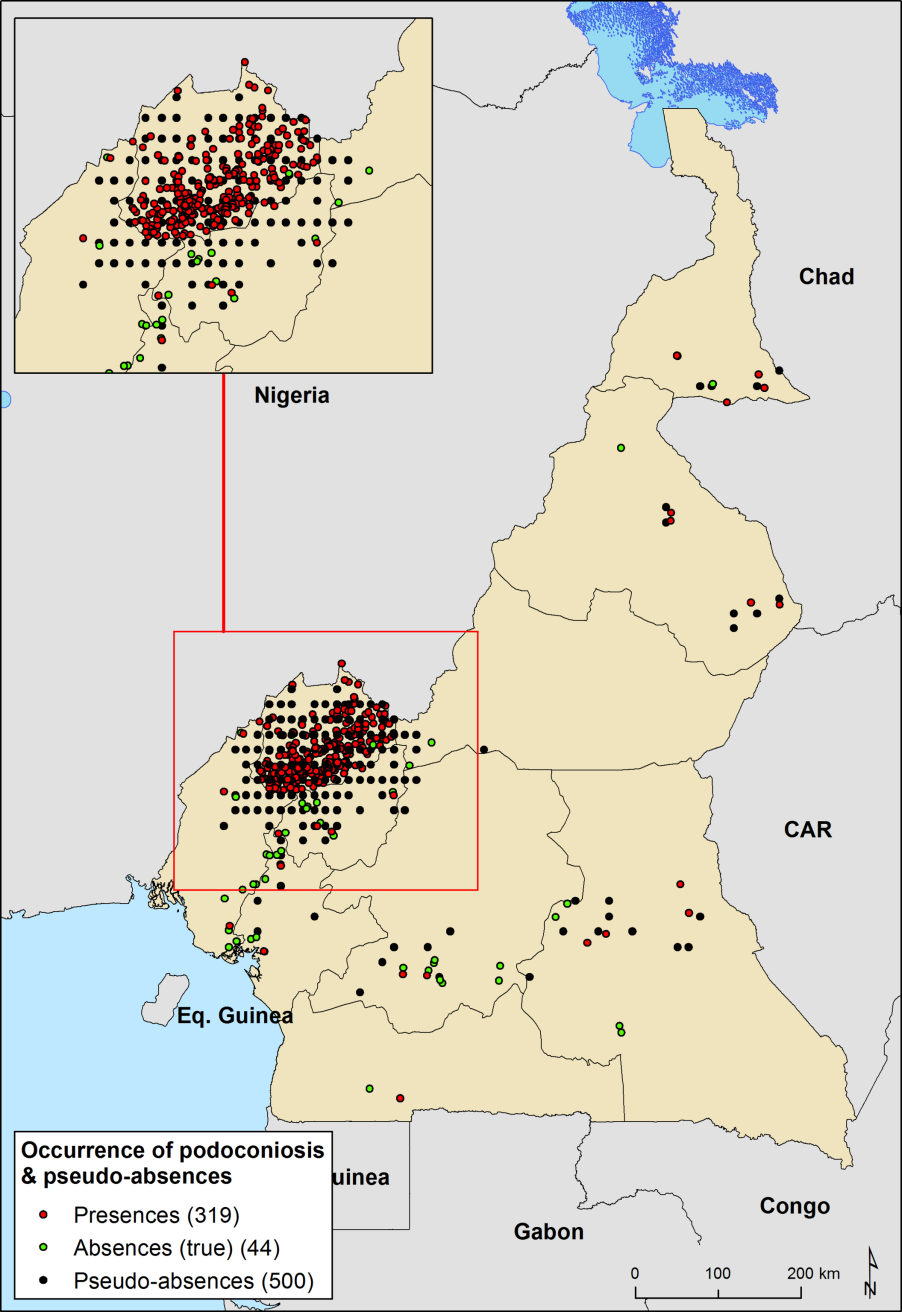
Distribution of surveyed community and background points for podoconiosis across Cameroon. Presence are points where presence of podoconiosis has been desired. Absence are areas where the absence of podoconiosis has been confirmed. Pseudo-absences are points to compensate for the lack of absence data; we created an evidence-based probabilistic framework for generating pseudo-absences.

We compiled a database of 748 geo-located prevalence records of podoconiosis in Cameroon (figure 1). Podoconiosis prevalence data were assembled from two cross-sectional surveys conducted in Cameroon. The first survey, conducted in the North West region of Cameroon in 2014, was a cross-sectional study involving stratified and cluster sampling. The sampling design and findings of this survey are detailed in a separate publication.^22^ Briefly, at least 50% of the communities from all the health areas in each of the 19 health districts of the region were screened for lymphoedema of the lower limbs. Preliminary community screening was carried out by trained community health implementers, and final confirmation of podoconiosis was done by expert research assistants and health personnel following a standardised clinical diagnosis algorithm.^21 23^ Overall, in the 19 Health Districts of the North West region of Cameroon, 204 551 individuals from 672 communities were investigated for podoconiosis. The second study was a nationwide cross-sectional survey conducted in 40 Health Districts from all 10 regions of Cameroon.^24^ In this survey, 76 communities were randomly selected, with 10 178 individuals from 4603 households screened for podoconiosis. Field workers used the same validated clinical diagnosis algorithm as that used in the first survey, to confirm podoconiosis case.

### Explanatory environmental variables

Data on extrinsic determinants of podoconiosis were assembled from remotely sensed environmental datasets (online supplementary figure 1S). Geographical coordinates of each community were used to extract from gridded map estimates on silt and clay soil fraction, pH of the soil, slope, precipitation, elevation, land surface temperature, distance to stable lights, EVI and distance to water surfaces (water bodies and streams).

10.1136/bmjgh-2018-000730.supp1 Supplementary file 1

Gridded continuous maps, namely raster datasets, of averaged EVI and land surface temperature (LST) for the period 2000–2015 were obtained from the African Soil Information System project.^25^ This project generates time series average products for several environmental indicators such as vegetation indices and LST using MODIS satellite image data collected by the National Aeronautics and Space Administration. The MOD13Q1 product from MODIS library, which is updated every 16 days at 250 m spatial resolution, includes vegetation indices such as Normalised Difference Vegetation Index and EVI^26^ day and night LST data are generated from MOD11A2 products, and have a spatial and temporal resolution of 1 km and 8 days, respectively.^27^

Information on rainfall was extracted from a synoptic gridded map of annual precipitation calculated from monthly total precipitation gridded datasets obtained from WorldClim database.^28^ This database provides a set of global climate layers obtained by interpolation of precipitation data for the period 1950–2000 collected in weather stations distributed across the world.^29^ From the Consortium for Spatial Information (CGIAR-CSI), we obtained a raster dataset of elevation at 1 km^2^.^30^ This elevation layer resulted from processing and resampling the gridded digital elevation models (DEM) derived from the original 30-arcsecond DEM produced by the Shuttle Radar Topography Mission. The elevation raster was processed to calculate terrain slope in degrees.

Soil data including silt and clay fraction and soil pH of the top soil were obtained from the ISRIC-World Soil Information project.^31^ This project provides gridded maps of soil composition at 250 m resolution worldwide. We also generated continuous surfaces of straight line distance (Euclidean distance) in kilometres to the nearest water body and permanent rivers based on the Global Database of Lakes, Reservoirs and Wetlands^32^ and Digital Global Chart,^33^ respectively.

Finally, night-light emissivity for 2013 captured by the Operational Linescan System instrument on board a satellite of the Defence Meteorological Satellite Programme was used as a proxy measure of poverty across Cameroon.^34^ This instrument measures visible and infrared radiation emitted at night time, resulting in remote imagery of lights on the ground. This information has been correlated with gross domestic product in developed countries^35 36^and, although far from precise, would provide an indirect measure of poverty in these areas.^37^

Input grids were resampled to a common spatial resolution of 1 km^2^ using the nearest-neighbour approach and clipped to match the geographical extent of a map of Cameroon, and eventually aligned to it. Raster manipulation and processing was undertaken using *raster* package in R V.3.3.2 and final map layouts created with ArcGIS V.10.3 software (ESRI, Redlands, CA, USA).

### Environmental modelling using machine learning approaches

An ensemble of distribution models was generated based on the reported occurrence of podoconiosis in the surveyed communities and the environmental factors. Communities were reclassified as endemic (1) or non-endemic (0) for podoconiosis based on records of confirmed podoconiosis cases. We used two machine learning-based algorithms available within the BIOMOD framework^38^ to obtain those ensembles of predicted distribution: generalised boosted regression tree modelling (BRT) and random forest (RF). The latter was run using the parameters set by default in the *biomod2* R package^38^ whereas for the former, the learning rate (*lr)* and tree complexity (*tc*), key parameters in BRT models, were set enabling the model to account for up to four potential interactions and slowing it down enough (*lr*: 0.005) to get the model converged without overfitting the data. This tuning was undertaken using the *gbm* package in R V.3.3.2.

All these models are intended to discriminate the suitability of the environment for the presence of podoconiosis (ie, environmental suitability), and for this they need to be trained with presence and absence records. From this first modelling exercise, we had to make some decisions regarding the community data to be used due to differences on sampling design between the two cross-sectional surveys. While the first survey dataset was obtained during an intensive screening exercise in a region known to be endemic for podoconiosis, the nationwide cross-sectional survey was intended to be geographically representative of disease distribution across the country. Therefore, the unbalanced representation of communities at North West region was compensated by selecting a random subset (75%) of ‘positive’ communities (reporting podoconiosis cases) from this region and generating a set of background points or pseudo-absences^39^ for the whole dataset (figure 1). Background points were randomly selected with the underlying geographical bias as the occurrence data, as some authors have recommended it.^40^ For this, we created a sampling bias surface by counting the number of occurrence records within each grid cell (1 km x 1 km resolution) and then extrapolated these data across Cameroon using kernel density estimation. We used *kernlab*, *ks* and *sm* R packages for running this process. Lastly, we generated the background points (n=500) from random locations weighted by the sampling bias surface.^41 42^ In order to maximise the ability of the model to discriminate between suitable and unsuitable areas, regression weights were used to down-weight pseudo-absence records, so that the summed weights of the absence and pseudo-absence records matched that of the presence records.

Models were calibrated using an 80% random sample of the initial data and evaluated against the remaining 20% data using the area under the curve (AUC) of the receiver operation characteristic (ROC), the true skill statistic (TSS)^43^ and the proportion correctly classified (PCC). Projections were performed 100 times, each time selecting a different 80% random sample while verifying model accuracy against the remaining 20%. The evaluation statistics (AUC and TSS) were used to select the models to be assembled based on the matching between predictions and observations. Here, models with AUC <0.8 or TSS values <0.7 were disregarded when assembling the final model.

The final assemble model was obtained by estimating the mean of probabilities across the selected models per grid cell. The range of uncertainties was also calculated by estimating the CIs around the mean of probabilities across the ensemble per grid cell.

The resulting predictive map quantifies the environmental suitability for podoconiosis. In order to convert this continuous metric into a binary map outlining the distribution limits (ie, ecological limits), a threshold value of suitability was determined, above which transmission was assumed to be possible. Based on the ROC curve, the threshold value that represents a better trade-off between sensitivity, specificity and PCC was determined.

In addition, partial dependence functions were performed separately for both modelling approaches (BRT and RF) to visualise dependencies between the probability of podoconiosis occurrence and covariates. The partial dependence function shows the marginal effect of each covariate on the response after averaging the effects of all other covariates.

### Geostatistical modelling to estimate disease burden

**Figure 2:**
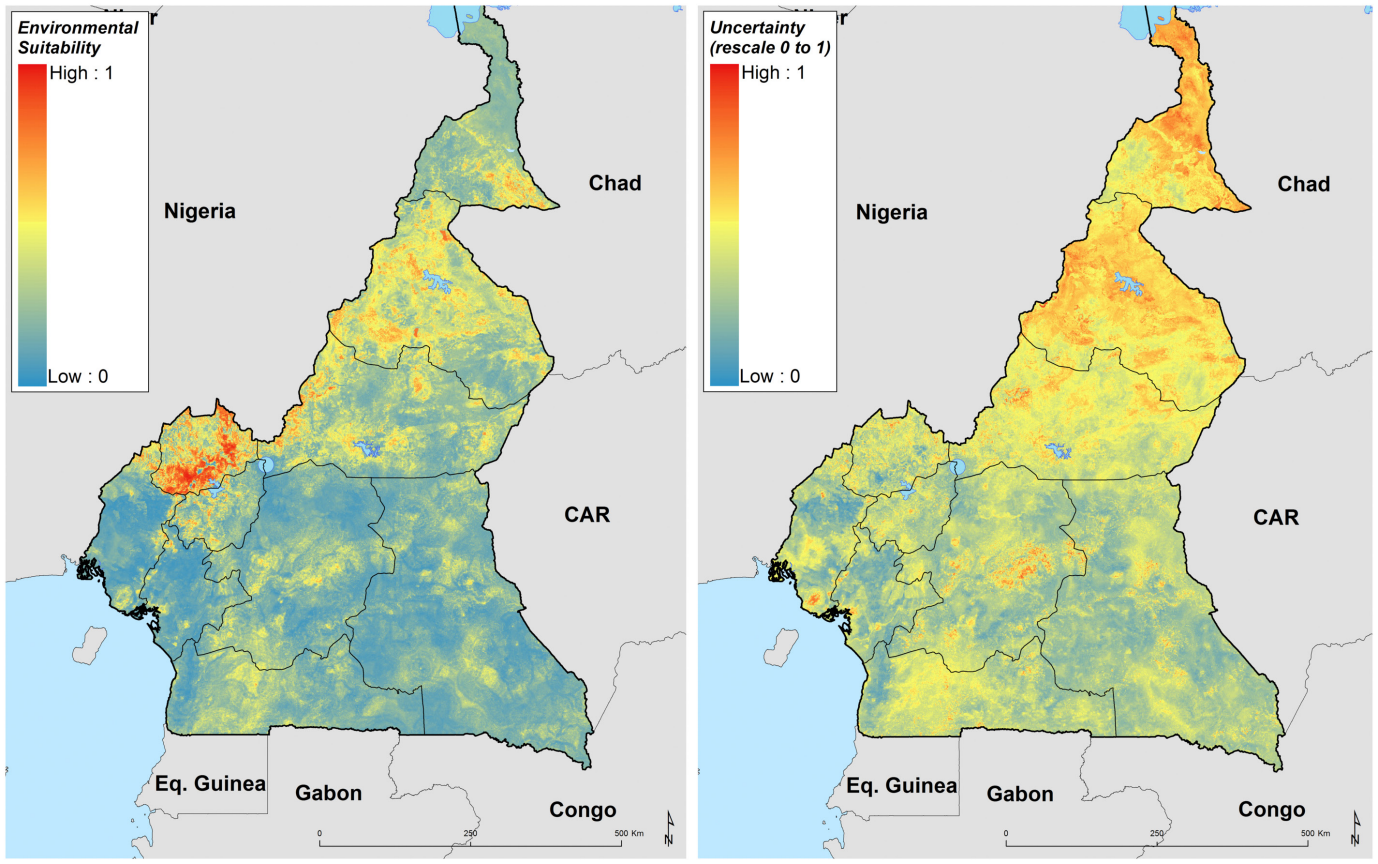
Ensemble of predicted environmental suitability models for podoconiosis and corresponding uncertainty of prediction. Uncertainty was calculated as the range of the 95% CI in predicted probability of occurrence for each pixel and rescaling to a 0–1 scale.

Empirical data and spatially matched covariates were then used within a geostatistical framework. We developed a geostatistical model to predict podoconiosis prevalence in environmentally suitable areas, as delineated by first modelling exercise, at village level across Cameroon. We let podoconiosis risk depend on the suite of measured risk factors mentioned above. We included spatial random effects in order to account for spatial variation in podoconiosis prevalence between villages that is not explained by the explanatory variables. We carried out validation of the model using a variogram-based procedure, which tests the compatibility of the adopted spatial structure with the data. More details are provided in the online supplementary material (Text 1S). The analysis was carried out using the R package *PrevMap*, which implements parameter estimation and spatial prediction of geostatistical models. This model was applied to produce continuous predictions of prevalence of podoconiosis among adults (≥15 years old) at 1 km^2^ spatial resolution and probability maps exceeding a 1% prevalence threshold, which were used to define podoconiosis endemicity. We checked the validity of the assumed covariance model for the spatial correlation using the Monte Carlo algorithm and empirical semi-variogram as described in the online supplementary file (figure 2S). Additionally, maps of the number of SEs from the posterior mean prevalence of podoconiosis (≥15 years) and number of cases were generated for each 1 km×1 km grid location.

Gridded maps of both population density and age structure were obtained from the WorldPop project.^44 45^ We used these gridded surfaces of population estimates to compute the potential affected adult population (older than 15). An output raster dataset computing the estimated number of podoconiosis cases per grid cell was obtained by multiplying the 1 km^2^ raster dataset of predictive prevalence with the corresponding adult population density surface. The same procedure was used to estimate the uncertainty range of the affected population using the gridded surfaces of 95% CI for predicted prevalence. These surfaces were then used to extract the aggregate number of people with podoconiosis and uncertainty range by administrative area (health districts and regions).

## Results

### Main outcomes of surveys

A total of 214 729 records of individuals screened for podoconiosis in 748 clusters were assembled for the current analysis from all 10 regions of Cameroon. Of the 214 729 screened individuals, 882 (0.4%; 95% CI 0.38 to 0.44) had podoconiosis. Of the 748 clusters, 59.2% (443/748) recorded zero cases of podoconiosis. On average, the number of individuals screened per cluster was 273, with 83% screening 100 or more individuals (table 1).

**Table 1:** General description of podoconiosis surveys conducted in Cameroon in 2014 and 2017

Region	Clusters surveyed	Total surveyed	Podoconiosis cases
Adamawa	2	320	0
Central	10	1932	4
East	8	1195	4
Extreme North	5	803	5
Littoral	9	1228	4
North	5	692	7
North West	681	205 664	849
South	2	435	1
South West	14	1137	3
West	12	1323	5
Total	748	214 729	882

### Factors associated with podoconiosis occurrence

**Figure 3:**
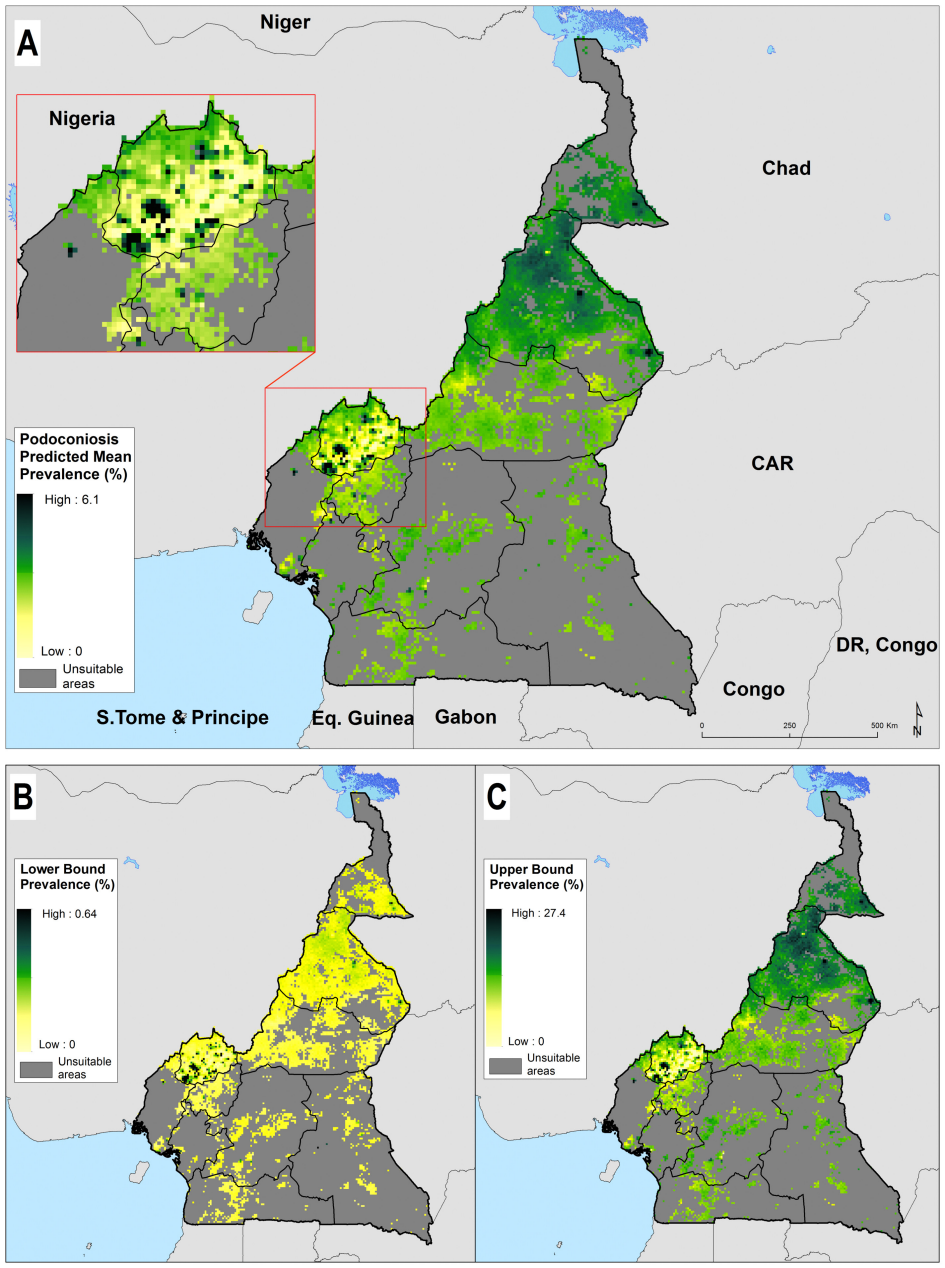
Predicted podoconiosis prevalence maps of Cameroon; mean predicted prevalence (A) and lower (B) and upper 95% CI bounds (C).

**Figure 4:**
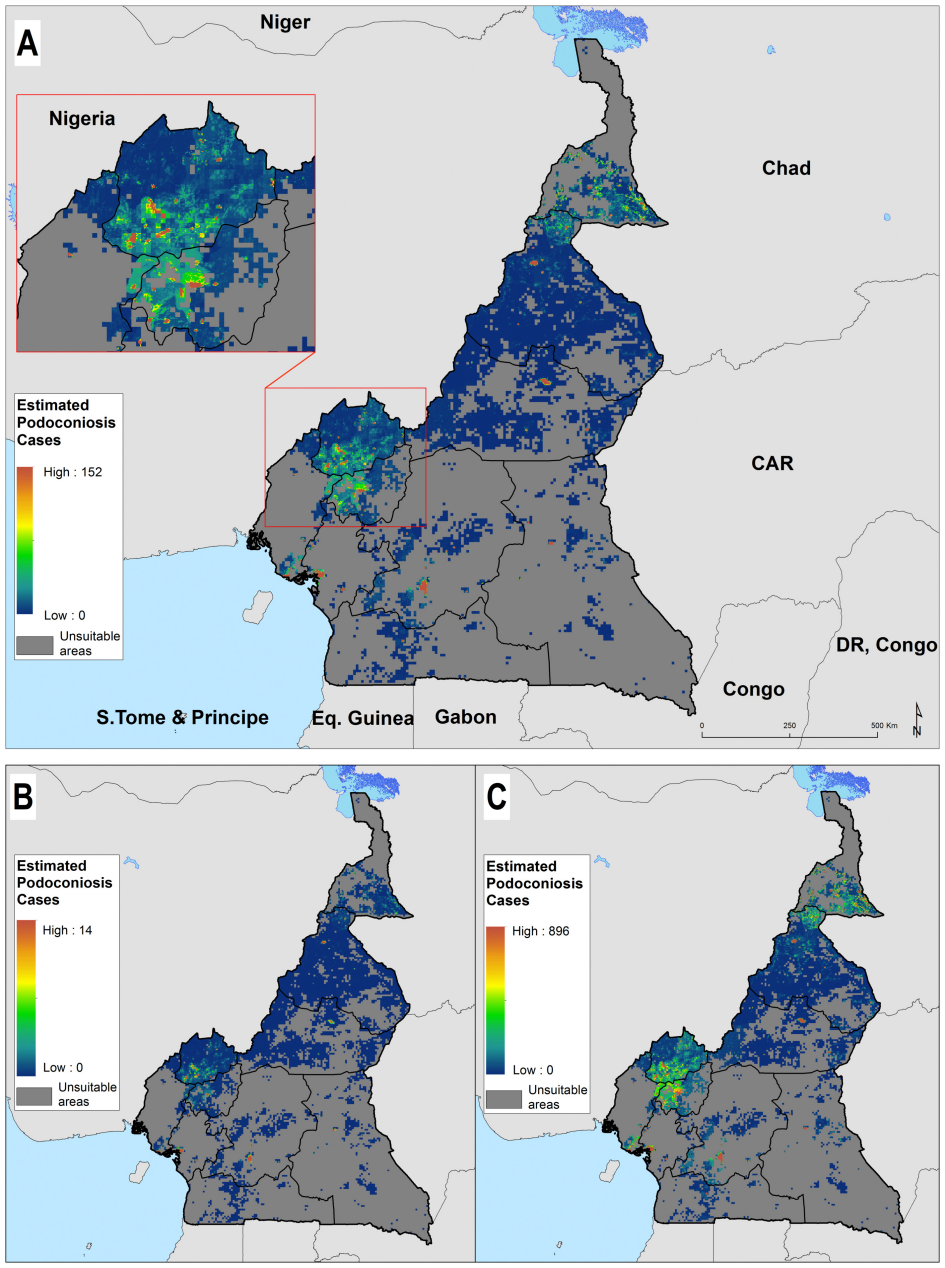
Estimated number of adults (≥15 years old) with podoconiosis across Cameroon: estimated number of cases (A) and lower (B) and upper 95% CI bounds (C).

**Figure 5:**
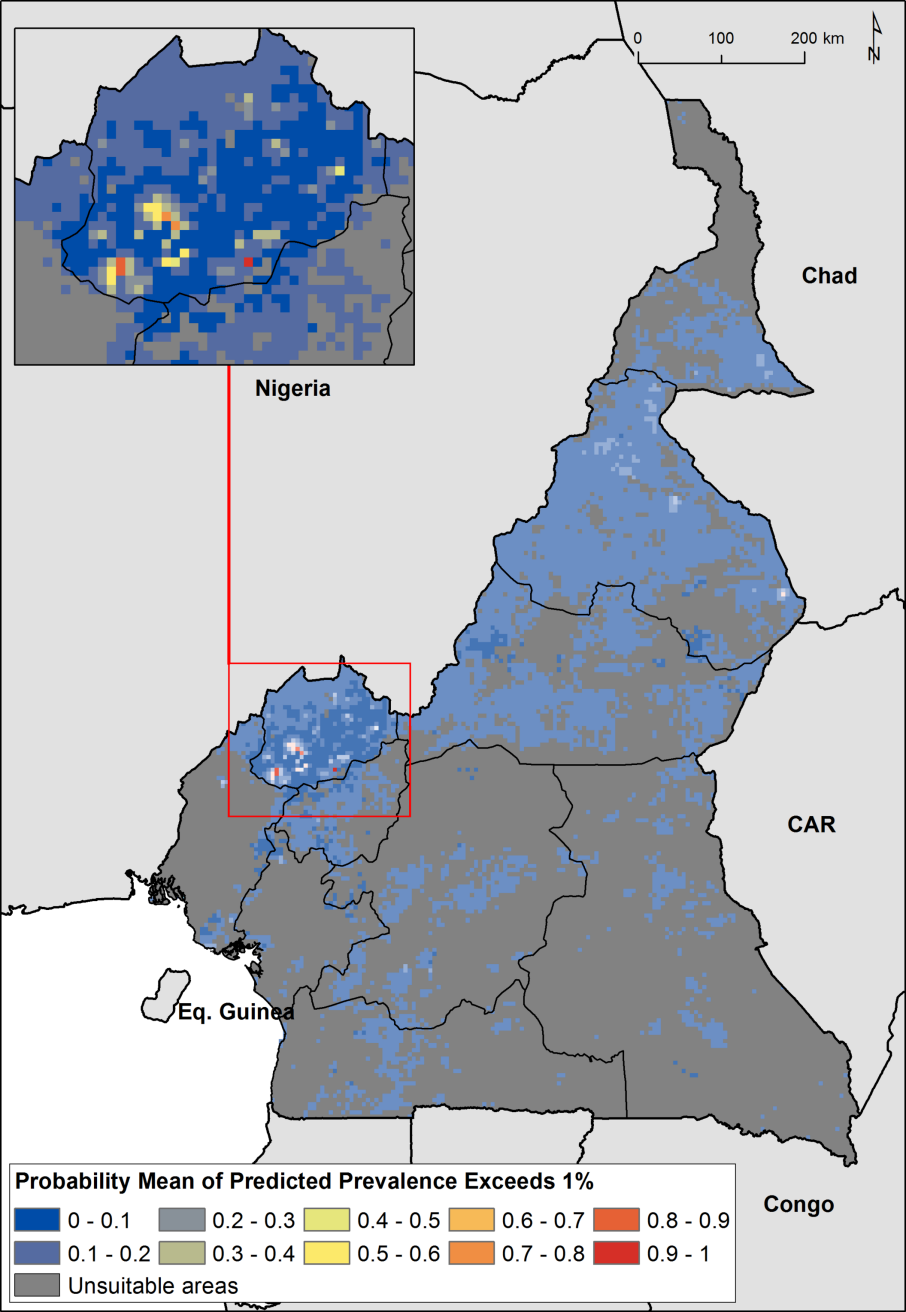
Map of probability of exceeding 1% podoconiosis prevalence in Cameroon.

Figures 3S to 6S in the online supplementary file show the marginal effect of each covariate on the probability of podoconiosis occurrence, while the relative contribution of each predictor variable on the outcome (podoconiosis prevalence) is summarised in figure 7S (online supplementary file). Both marginal effect plots and covariate contribution have been estimated separately for BRT and RF assemble models. Briefly, 6 of 11 selected environmental covariates were the major contributors to the assemble models: silt and clay fraction of top soil, precipitation, elevation, slope and distance to stable night lights (online supplementary figure 5S). In both modelling approaches, when the silt fraction exceeds 25%, the probability of podoconiosis occurrence increases. The association of probability of podoconiosis and annual precipitation is steadily high over 1000 mm and sharply decreases when the annual mean rainfall goes beyond 2000 mm to 2500 mm. Areas located between 1000 masl (metres above sea level) and 2000 masl are most suitable for the occurrence of podoconiosis. Slope above 10 degrees and clay fraction of the top soil exceeding 40% seem to prevent the occurrence of podoconiosis (online supplementary figure 3S to 6S).

### Environmental limits of podoconiosis in Cameroon

High environmental suitability for podoconiosis was predicted in three Regions of Cameroon (Adamawa, North West and North). Absence of podoconiosis was predicted in much of South West, Littoral, East, Central and South regions (figure 2). A suitability cut-off of 0.43 (0.39 to 0.45, for 95% CI lower and upper bounds, respectively) with a sensitivity of 99.6% and specificity 99.8% provided the best discrimination between presence and absence records in the training data, and therefore this threshold value was used to reclassify the predictive risk map into a binary map outlining the potential environmental limits of occurrence (figure 8S, online supplementary file). Uncertainty was calculated as the range of the 95% CI in predicted probability of occurrence for each pixel (figure 2) indicating high uncertainty in the northern part of the Extreme North region. Cross-validation analysis for the BRT and RF ensemble models using a 20% held-out subsample indicated their high predictive performance, with AUC values of 0.92 (95% CI 0.9 to 0.94) and 0.96 (95% CI 0.95 to 0.97), respectively. This high performance is also consistent through the true skill statistic, with TSS values of 0.78 (95% CI 0.75 to 0.82) and 0.82 (95% CI 0.8 to 0.86) for the BRT and RF models, respectively.

### Predicted prevalence, population at risk and estimation of podoconiosis burden

The national population living in areas environmentally suitable for podoconiosis is estimated to be over 5.2 (95% CI 4.7 to 5.8) million, which corresponds to 22.3% of Cameroon’s population in 2015. The largest portion (32.2%) of the most-at-risk population live in the North West Region.

The predicted prevalence map showed heterogeneous distribution of podoconiosis burden across Cameroon (figure 3). The highest prevalence of podoconiosis is predicted in four regions (Adamawa, North West, North and in some parts of Extreme North). In the remaining regions, the distribution of podoconiosis would be focal and prevalence low. Nationally, we estimated 41 556 adults (95% CI 1170 to 240 993) to be living with podoconiosis in 2015 in Cameroon (table 2). Four regions (Central, Littoral, North and North West) contributed 61.2% of the absolute number of cases (figure 4). The greatest proportion of all individuals with podoconiosis resided in the Central Region (17.6%). The South and East regions contributed marginally to the total number of people with podoconiosis. At least one case of podoconiosis was estimated in 170 of 189 Health Districts. A total of 94 Health Districts reported ≥100 podoconiosis cases and only 20 had more than 500 predicted cases (table 1S). We have also estimated the continuous probability of exceeding 1% podoconiosis prevalence (the threshold considered for intervention) across the endemic areas (figure 5). Most of the areas have low probability of exceeding 1%, and only a few restricted areas at the North West region would potentially exceed that threshold.

**Table 2:** Estimated number of podoconiosis cases and population at risk among adults in Cameroon in 2015.

	Estimated population at risk			Estimated podoconiosis burden		
Regions	Population estimates	Lower bound	Upper bound	Adult estimated cases	Lower bound	Upper bound
Adamawa	381 666	361 913	420 270	2305	49	13 831
Central	400 747	306 628	431 475	7303	176	43 138
East	80 736	72 706	97 824	899	22	5293
Extreme North	547 793	493 820	613 170	5134	112	30 902
Littoral	618 549	491 969	749 893	6186	237	34 237
North	595 335	504 622	757 766	5840	128	35 152
North West	1 678 461	1 649 810	1 719 003	6089	271	32 011
South	126 695	120 569	131 644	840	19	5043
South West	203 965	193 811	229 278	2521	59	14 867
West	583 260	546 435	652 894	4441	99	26 519
Grand total	5 217 208	4 742 282	5 803 216	41 556	1170	240 993

## Discussion

Podoconiosis is a highly neglected disease that is often under-reported in endemic countries.^2 46^ Understanding the occurrence of podoconiosis is crucial for identifying populations at risk and to estimate the number of cases in order to scale up interventions.^2^ Here, we used data on podoconiosis prevalence to model the environmental suitability, estimate the population at risk and the number of cases of podoconiosis in Cameroon. We quantified the relationship of climate, environmental and meteorological factors to the spatial distribution of podoconiosis. Our model prediction suggests marked ecological limits separating the broad areas of environmental suitability in western, central and northern parts of Cameroon from the southern and eastern parts of the country, which are considered to be free of podoconiosis. Despite estimating a large number of individuals living in the predicted podoconiosis risk zone (5.2 million), the total number potentially affected would be relatively small (41 556 adults). This suggests that the disease could be controlled and eliminated in Cameroon if the appropriate interventions were put in place in the most-at-risk areas. However, current intervention efforts in the country cover only a fraction of the population potentially at risk.^2^

We believe this work increases insight into the epidemiology of podoconiosis and simultaneously has practical consequences for the Cameroon health system. First, the identification of areas at risk and quantification of disease burden presented in this work should support more comprehensive plans for podoconiosis control in Cameroon. The risk maps presented can help set priority areas for intervention and lead to more rational use of available resources. Health services and surveillance systems in these at-risk areas should be prepared to diagnose podoconiosis cases correctly and provide the necessary healthcare that patients require. Ensuring health workers are well trained in the diagnosis and management of podoconiosis is essential.

Second, we have extended the understanding of the environmental drivers of podoconiosis. In addition to the factors identified in previous work,^13 24 46^ such as precipitation, elevation and soil composition, we have found that land surface temperature, distance from stable night-light and pH of the soil may contribute to the risk of podoconiosis occurrence. The results here indicate that, although the same suite of environmental and climatic factors drive the distribution of podoconiosis in different settings, there is spatial variation in their effect and relative contribution. The interplay among podoconiosis risk, climate, environment and socioeconomic development is inevitably complex. Our analysis highlights the fact that a focus on simple, single factors fails to adequately explain the risk of podoconiosis. This study provides an analytical framework for developing podoconiosis risk model and estimating the disease burden in other potentially endemic countries. Ultimately, it will also contribute to the construction of constructing continental and global risk maps, as well as an and to estimation of the actual global burden of podoconiosis at global scale.^5^

In the current analysis, we have identified environmental factors associated with the risk of occurrence of podoconiosis. In addition to the factors identified in our previous model in Ethiopia, we have identified^13 14 47^ land surface temperature, distance to stable night-lights and pH of the soil as additional factors that drive the risk of occurrence of podoconiosis. In our previous model using data from Ethiopia, the factors that were found to be associated with the spatial distribution of podoconiosis were elevation, precipitation and EVI.^13 14 47^ According to our study, major drivers of podoconiosis occurrence in Cameroon appear to be soil composition and landscape characteristics (silt and clay composition of soil and slope of the land).

Our results show that podoconiosis is present in other regions of Cameroon besides the historical endemic North West region.^24^ Most of the areas where high prevalence of podoconiosis is documented are in the Cameroon Volcanic Line (CVL).^48^ The CVL is a 1600 km chain of volcanoes that extends along the border region of eastern Nigeria and includes islands such as São Tomé and Príncipe,^49^ and Bioko,^18^ which are also endemic for podoconiosis. These volcanic activities occurred over 1 million years ago,^48^ with subsequent weathering and generation of soils. Dense tropical forest has flourished over these rich soils for thousands of years and become highly rich in clay and silt because of the decomposition of organic matter.

Although podoconiosis was reported four decades ago in Cameroon,^19 50^ there has been no systematic effort to map the distribution and quantify the burden in the country. Podoconiosis services are only available in the North West region provided by a non-governmental organisation. Exposure to soil is common, as shoes are not worn regularly in rural areas of Cameroon.^50^ They are preserved for special occasions such as attending weddings, church ceremonies and weekly markets.^51^ In a survey conducted in the North West region, only a handful of interviewees reported wearing shoes during farm-related activities such as planting, harvesting and working on a rice farm.^22^ This is likely to have contributed to the continued presence of the disease in environmentally suitable areas. The lack of attention by national agencies and international partners, and difficulties in ensuring access to preventive methods, such as shoes,^52^ are listed among the major challenges for podoconiosis elimination.

This study had some limitations. First, most of the prevalence data gathered for this study came from the North West region of the country, which may have introduced geographical bias in the analysis. We mitigated the impact of this bias by generating more random background points around areas with more dense distribution of communities reporting podoconiosis cases. Furthermore, the nationwide mapping survey was designed to capture the potential variation of podoconiosis risk across the country. Thus, although sparse, surveyed communities were selected from the various ecological settings existing in the country. Second, although we accounted for the most significant environmental predictors when constructing our models, we did not include important risk factors that operate at the individual or household level, such as shoe-wearing practice and household socioeconomic status.^13 15 53^ We tried to minimise this limitation by including a proxy measure of poverty (night-light emissivity).^37^ However, the wide CIs around the estimates of prevalence and disease burden point to important risk factors, which we may not yet be taking into account. Third, the results also indicate that the unexplained variation on scale smaller than 1.2 km is the main contributor to total variation in podoconiosis prevalence. In statistical terms, this is reflected by large estimates for the variance of unstructured random effect (also known as nugget effect), which incorporates the cumulative effect of both individual and within-communities unmeasured risk factor. Finally, larger sample size would have led to more precise predication, but the sample size used was the largest we could achieve given the available resources.

This is the first comprehensive assembly of contemporary data on podoconiosis occurrence and prevalence in Cameroon. We have applied new modelling approaches to maximise the predictive power of these data.^54 55^ However, data on podoconiosis are scant both in space and time compared with other diseases and neglected tropical diseases, such as soil-transmitted helminth infections,^56 57^ malaria^58^ and lymphatic filariasis.^59^ The production of fine-resolution maps of podoconiosis is contingent on the availability of geo-referenced data. A simplified case definition of podoconiosis can be included in the integrated diseases surveillance systems in endemic countries to heighten the index of suspicion among healthcare providers.

## Conclusion

The distribution of podoconiosis in Cameroon is wider than initially thought, according to our predictive models. The number of cases and population at risk are considerable in Cameroon. The findings presented here indicate the need to scale up interventions for those at risk and in need of care services. Promotion of footwear and foot hygiene through social mobilisation will be important. Morbidity management and disability prevention services should be made accessible to those suffering from the condition. The results presented here may help decision-makers to make evidence-based plans and evaluate performance.

## References

[1] United to combat neglected tropical diseases. 12 2017 Reaching a Billion. Ending Neglected Tropical Diseases: A gateway to Universal Health Coverage Fifth progress report on the London Declaration on NTDs. http://unitingtocombatntds.org/wp-content/themes/tetloose/app/staticPages/fifthReport/files/fifth_progress_report_english.pdf Available at Accessed on 2 April, 2018.

[2] Deribe K, Tekola-Ayele F, Davey G Podoconiosis: Endemic non-filarial elephantiasis : Gyapong J, Boatin B, *Neglected Tropical Diseases*—*Sub-Saharan Africa 1st ed 2016 Edition*. Switzerland: Springer International Publishing, 2016:231–49.

[3] Molyneux DH Tropical lymphedemas—control and prevention. N Engl J Med 2012;366:1169–71. 10.1056/NEJMp1202011 22455411

[4] Tekola Ayele F, Adeyemo A, Finan C, et al HLA class II locus and susceptibility to podoconiosis. N Engl J Med 2012;366:1200–8. 10.1056/NEJMoa1108448 22455414 PMC3350841

[5] Deribe K, Cano J, Newport MJ, et al The global atlas of podoconiosis. Lancet Glob Health 2017;5:e477–e479. 10.1016/S2214-109X(17)30140-7 28395836 PMC5390851

[6] WHO. Lymphatic filariasis: Podoconiosis: endemic non-filarial elephantiasis. 2017 http://www.who.int/lymphatic_filariasis/epidemiology/podoconiosis/en/ (accessed 15 Dec 2017).

[7] Deribe K, Cano J, Giorgi E, et al Estimating the number of cases of podoconiosis in Ethiopia using geostatistical methods. Wellcome Open Res 2017;2:78 10.12688/wellcomeopenres.12483.1 29152596 PMC5668927

[8] Deribe K, Kebede B, Tamiru M, et al Integrated morbidity management for lymphatic filariasis and podoconiosis, Ethiopia. Bull World Health Organ 2017;95:652–6. 10.2471/BLT.16.189399 28867846 PMC5578380

[9] Deribe K, Wanji S, Shafi O, et al The feasibility of eliminating podoconiosis. Bull World Health Organ 2015;93:712–8. 10.2471/BLT.14.150276 26600613 PMC4645432

[10] Sikorski C, Ashine M, Zeleke Z, et al Effectiveness of a simple lymphoedema treatment regimen in podoconiosis management in southern Ethiopia: one year follow-up. PLoS Negl Trop Dis 2010;4:e902 10.1371/journal.pntd.0000902 21152059 PMC2994920

[11] Negussie H, Molla M, Ngari M, et al Lymphoedema management to prevent acute dermatolymphangioadenitis in podoconiosis in northern Ethiopia (GoLBeT): a pragmatic randomised controlled trial. Lancet Glob Health 2018 (Epub ahead of print 14 May). 10.1016/S2214-109X(18)30124-4 PMC6562300 29773516

[12] Deribe K, Brooker SJ, Pullan RL, et al Spatial distribution of podoconiosis in relation to environmental factors in Ethiopia: a historical review. PLoS One 2013;8:e68330 10.1371/journal.pone.0068330 23874587 PMC3706425

[13] Deribe K, Brooker SJ, Pullan RL, et al Epidemiology and individual, household and geographical risk factors of podoconiosis in Ethiopia: results from the first nationwide mapping. Am J Trop Med Hyg 2015;92:148–58. 10.4269/ajtmh.14-0446 25404069 PMC4288951

[14] Deribe K, Cano J, Newport MJ, et al Mapping and modelling the geographical distribution and environmental limits of podoconiosis in Ethiopia. PLoS Negl Trop Dis 2015;9:e0003946 10.1371/journal.pntd.0003946 26222887 PMC4519246

[15] Molla YB, Wardrop NA, Le Blond JS, et al Modelling environmental factors correlated with podoconiosis. Int J Health Geogr 2014;13:24. 24946801 10.1186/1476-072X-13-24 PMC4082615

[16] Price EW The association of endemic elephantiasis of the lower legs in East Africa with soil derived from volcanic rocks. Trans R Soc Trop Med Hyg 1976;70:288–95. 10.1016/0035-9203(76)90078-X 1006757

[17] Price EW, Bailey D Environmental factors in the etiology of endemic elephantiasis of the lower legs in tropical Africa. Trop Geogr Med 1984;36:1–5. 6328708

[18] Price E Podoconiosis: Non-filarial Elephantiasis. Oxford, UK: Oxford Medical Publications, 1990.

[19] Price EW, Henderson WJ Endemic elephantiasis of the lower legs in the United Cameroon Republic. Trop Geogr Med 1981;33:23–9. 7245337

[20] Wanji S, Tendongfor N, Esum M, et al Elephantiasis of non-filarial origin (podoconiosis) in the highlands of north-western Cameroon. Ann Trop Med Parasitol 2008;102:529–40. 10.1179/136485908X311849 18782492

[21] Wanji S, Kengne-Ouafo JA, Datchoua-Poutcheu FR, et al Detecting and staging podoconiosis cases in North West Cameroon: positive predictive value of clinical screening of patients by community health workers and researchers. BMC Public Health 2016;16:997 10.1186/s12889-016-3669-6 27650390 PMC5029032

[22] Wanji S, Kengne-Ouafo JA, Deribe K, et al Study of lymphoedema of non-filarial origin in the northwest region of Cameroon: spatial distribution, profiling of cases and socio-economic aspects of podoconiosis. Int Health 2018:2017 10.1093/inthealth/ihy028 PMC6038914 29771349

[23] Sime H, Deribe K, Assefa A, et al Integrated mapping of lymphatic filariasis and podoconiosis: lessons learnt from Ethiopia. Parasit Vectors 2014;7:397 10.1186/1756-3305-7-397 25164687 PMC4153915

[24] Deribe K, Andrew AB, Cano J, et al Mapping the geographical distribution of podoconiosis in Cameroon using parasitological, serological, and clinical evidence to exclude other causes of lymphedema. PLoS Negl Trop Dis. 2017 In Press. 10.1371/journal.pntd.0006126 PMC5764238 29324858

[25] Africa Soil Information System. http://www.africasoils.net/data/datasets (accessed 20 Jan 2014).

[26] DAAC NL. NASA LP DAAC: MOD13Q1 Vegetation Indices 16-Day L3 Global 250m: NASA EOSDIS Land Processes DAAC, USGS Earth Resources Observation and Science (EROS) Center, Sioux Falls, South Dakota. https://lpdaac.usgs.gov (accessed Jan 2017).

[27] DAAC NL. NASA LP DAAC: MOD11A2 Land Surface Temperature and Emissivity 8-Day L3 Global 1km: NASA EOSDIS Land Processes DAAC, USGS Earth Resources Observation and Science (EROS) Center, Sioux Falls, South Dakota. https://lpdaac.usgs.gov (accessed Jan 2017).

[28] WorldClim. Global Climate data. http://www.worldclim.org/

[29] Hijmans RJ, Cameron SE, Parra JL, et al Very high resolution interpolated climate surfaces for global land areas. Int J Climatol 2005;25:1965–78. 10.1002/joc.1276

[30] CGIAR-CSI. Consortium for Spatial Information. http://www.cgiar-csi.org/

[31] ISRIC - World Soil Information. Soil property maps of Africa at 1 km. http://www.isric.org (accessed 20 Jan 2014).

[32] Lehner B, Döll P Development and validation of a global database of lakes, reservoirs and wetlands. J Hydrol 2004;296:1–22. 10.1016/j.jhydrol.2004.03.028

[33] DIVA-GIS. Digital global chart: inland waters. http://www.diva-gis.org/gdata

[34] Elvidge CD, Baugh KE, Kihn EA, et al Mapping city lights with nighttime data from the DMSP operational linescan system. Photogramm Eng Rem S 1997;63:727–34.

[35] Doll CNH, Muller J-P, Morley JG Mapping regional economic activity from night-time light satellite imagery. Ecological Economics 2006;57:75–92. 10.1016/j.ecolecon.2005.03.007

[36] Ebener S, Murray C, Tandon A, et al From wealth to health: modelling the distribution of income per capita at the sub-national level using night-time light imagery. Int J Health Geogr 2005;4:5 10.1186/1476-072X-4-5 15705196 PMC549533

[37] Noor AM, Alegana VA, Gething PW, et al Using remotely sensed night-time light as a proxy for poverty in Africa. Popul Health Metr 2008;6:5 10.1186/1478-7954-6-5 18939972 PMC2577623

[38] Thuiller W, Lafourcade B, Engler R, et al BIOMOD—a platform for ensemble forecasting of species distributions. Ecography 2009;32:369–73. 10.1111/j.1600-0587.2008.05742.x

[39] VanDerWal J, Shoo LP, Graham C, et al Selecting pseudo-absence data for presence-only distribution modeling: how far should you stray from what you know? Ecol Modell 2009;220:589–94. 10.1016/j.ecolmodel.2008.11.010

[40] Phillips SJ, Dudík M, Elith J, et al Sample selection bias and presence-only distribution models: implications for background and pseudo-absence data. Ecol Appl 2009;19:181–97. 10.1890/07-2153.1 19323182

[41] Elith J, Kearney M, Phillips S The art of modelling range-shifting species. Methods Ecol Evol 2010;1:330–42. 10.1111/j.2041-210X.2010.00036.x

[42] Fitzpatrick MC, Gotelli NJ, Ellison AM MaxEnt versus MaxLike: empirical comparisons with ant species distributions. Ecosphere 2013;4:art55 10.1890/ES13-00066.1

[43] Liu C, White M, Newell G Measuring and comparing the accuracy of species distribution models with presence-absence data. Ecography 2011;34:232–43. 10.1111/j.1600-0587.2010.06354.x

[44] Tatem AJ, Noor AM, von Hagen C, et al High resolution population maps for low income nations: combining land cover and census in East Africa. PLoS One 2007;2:e1298 10.1371/journal.pone.0001298 18074022 PMC2110897

[45] Linard C, Gilbert M, Snow RW, et al Population distribution, settlement patterns and accessibility across Africa in 2010. PLoS One 2012;7:e31743 10.1371/journal.pone.0031743 22363717 PMC3283664

[46] Kihembo C, Masiira B, Lali WZ, et al Risk factors for podoconiosis: Kamwenge District, Western Uganda, September 2015. Am J Trop Med Hyg 2017;96:1490–6. 10.4269/ajtmh.16-0932 28719274 PMC5462591

[47] Diggle PJ, Thomson MC, Christensen OF, et al Spatial modelling and the prediction of Loa loa risk: decision making under uncertainty. Ann Trop Med Parasitol 2007;101:499–509. 10.1179/136485913X13789813917463 17716433

[48] De Plaen RSM, Bastow ID, Chambers EL, et al The development of magmatism along the Cameroon Volcanic Line: evidence from seismicity and seismic anisotropy. J Geophys Res 2014;119:4233–52. 10.1002/2013JB010583

[49] Ruiz L, Campo E, Corachán M Elephantiasis in São Tomé and Príncipe. Acta Trop 1994;57:29–34. 10.1016/0001-706X(94)90090-6 7942352

[50] Price EW, McHardy WJ, Pooley FD Endemic elephantiasis of the lower legs as a health hazard of barefooted agriculturalists in Cameroon, West Africa. Ann Occup Hyg 1981;24:1–8. 7235457 10.1093/annhyg/24.1.1

[51] Mukum JM Culture and Customs of Cameroon. Westport, Connecticut and London: Greenwood press, 2005.

[52] Ayode D, McBride CM, de Heer HD, et al A qualitative study exploring barriers related to use of footwear in rural highland ethiopia: implications for neglected tropical disease control. PLoS Negl Trop Dis 2013;7:e2199 10.1371/journal.pntd.0002199 23638211 PMC3636134

[53] Molla YB, Le Blond JS, Wardrop N, et al Individual correlates of podoconiosis in areas of varying endemicity: a case–control study. PLoS Negl Trop Dis 2013;7:e2554 10.1371/journal.pntd.0002554 24340109 PMC3854961

[54] De’ath G, De’ath G Boosted trees for ecological modeling and prediction. Ecology 2007;88:243–51. doi:10.1890/0012-9658(2007)88[243:BTFEMA]2.0.CO;2 17489472

[55] Giorgi E, Diggle PJ PrevMap: an *R* package for prevalence mapping. J Stat Softw 2017;78:1–29. 10.18637/jss.v078.i08

[56] Pullan RL, Brooker SJ The global limits and population at risk of soil-transmitted helminth infections in 2010. Parasit Vectors 2012;5:81 10.1186/1756-3305-5-81 22537799 PMC3419672

[57] Pullan RL, Smith JL, Jasrasaria R, et al Global numbers of infection and disease burden of soil transmitted helminth infections in 2010. Parasit Vectors 2014;7:37 10.1186/1756-3305-7-37 24447578 PMC3905661

[58] Hay SI, Snow RW The malaria Atlas Project: developing global maps of malaria risk. PLoS Med 2006;3:e473 10.1371/journal.pmed.0030473 17147467 PMC1762059

[59] Cano J, Rebollo MP, Golding N, et al The global distribution and transmission limits of lymphatic filariasis: past and present. Parasit Vectors 2014;7:466 10.1186/s13071-014-0466-x 25303991 PMC4197264

