# Supplementary material for: Predicted distribution and burden of podoconiosis in Cameroon
Source: BMJ Glob Health. 2018 Jun 22;3(3):e000730. doi: 10.1136/bmjgh-2018-000730 (PMC6014185; doi:10.1136/bmjgh-2018-000730)
Supplement: Supplementary file 1 [file bmjgh-2018-000730supp001.pdf]

Predicted distribution and burden of podoconiosis in Cameroon.

Supplementary file

Text 1S. Formulation and validation of geostatistical model of podoconiosis prevalence

Let Y_i denote the number of positively tested podoconiosis cases at location x_i out of n_i sample individuals. We then assume that, conditionally on a zero-mean spatial Gaussian process *S(x)*, the Y_i are mutually independent Binomial variables with probability of testing positive $p(x_i)$ such that

$$log\left\{\frac{p(x_i)}{1-p(x_i)}\right\} = \beta_0 + \beta_1 Clay(x_i) + \beta_2 DSTL(x_i) + \beta_3 DSTW(x_i) + \beta_4 E(x_i) + \beta_5 Prec(x_i) + \beta_6 Silt(x_i) + S(x_i)$$

where the explanatory in the above equation are, in order, fraction of clay, distance (in meters) to stable light (DSTL), distance to water bodies (DSTW), elevation (E), precipitation(Prec) (in mm) and fraction of silt at location x_i.

We model the Gaussian process *S(x)* using an isotropic and stationary exponential covariance function given by

$$Cov\{S(x), S(x')\} = \sigma^2 exp\{-||x-x'||/\phi\}$$

Where $||x-x'||$is the Euclidean distance between *x* and *x'*, σ^2 is the variance of *S(x)* and ϕ is a scale parameter that regulates how fast the spatial correlation decays to zero for increasing distance.

To check the validity of the adopted exponential correlation function for the spatial random effects *S(x)*, we carry out the following Monte Carlo algorithm.

1. Simulate a binomial geostatistical data-set at observed locations x_i by plugging-in the maximum likelihood estimates from the fitted model.
2. Estimate the unstructured random effects Z_i from a non-spatial binomial mixed model obtained by setting *S(x) =0* for all locations *x*.
3. Use the estimates for Z_i from the previous step to compute the empirical variogram.
4. Repeat steps 1 to 3 for 10,000 times.
5. Use the resulting 1,000 variograms to compute the 95% tolerance bandwidth under the hypothesis that the analysed data were generated by the fitted model. If the empirical variogram from the original data, obtained as in step 2, lies within 95% bandwidth, we then conclude that we do not find evidence against the assumption of an exponential correlation function for *S(x)*.

Predicted distribution and burden of podoconiosis in Cameroon.

Figure 1S. Maps of covariates used to model environmental suitability and podoconiosis prevalence.

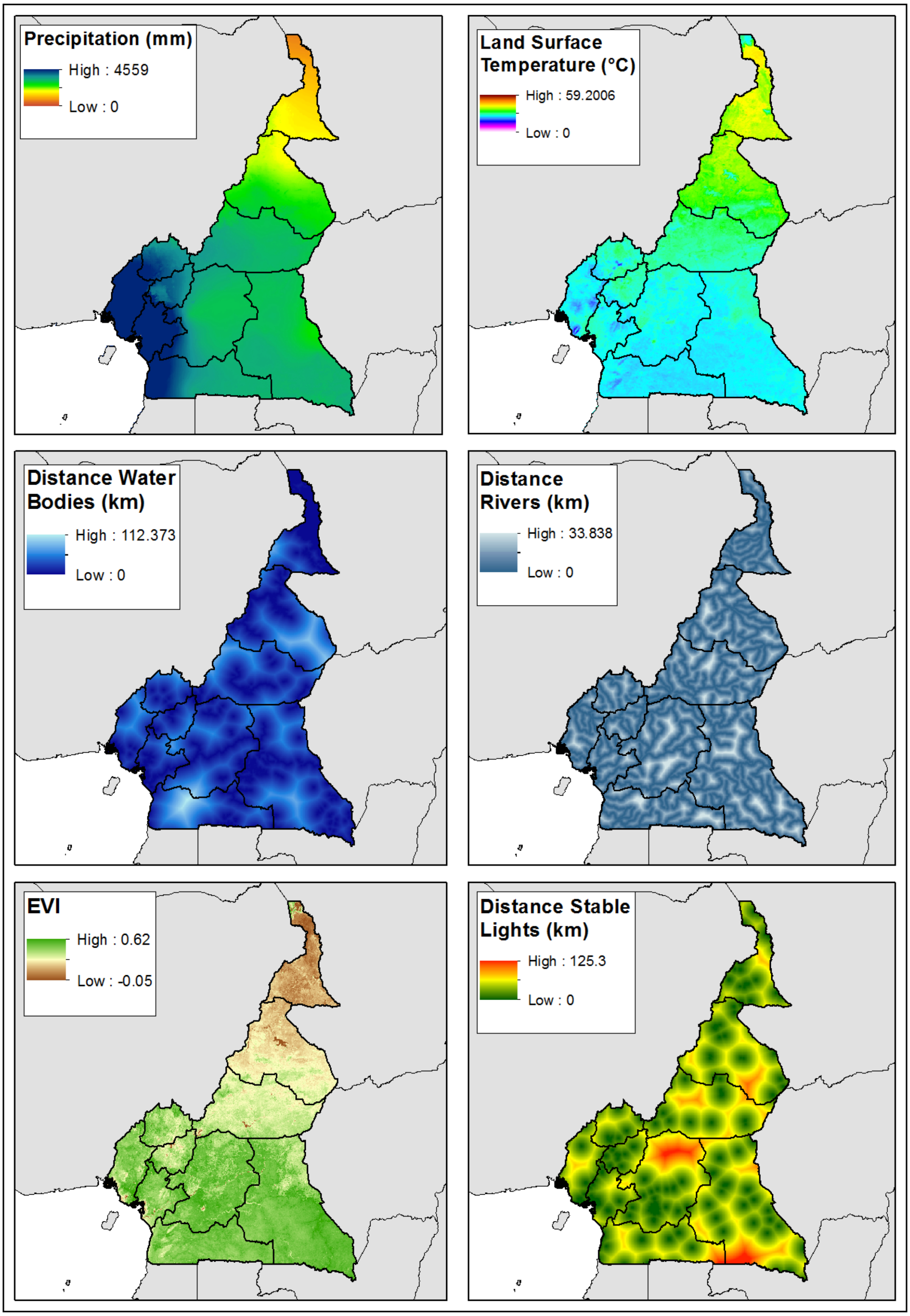

Predicted distribution and burden of podoconiosis in Cameroon.

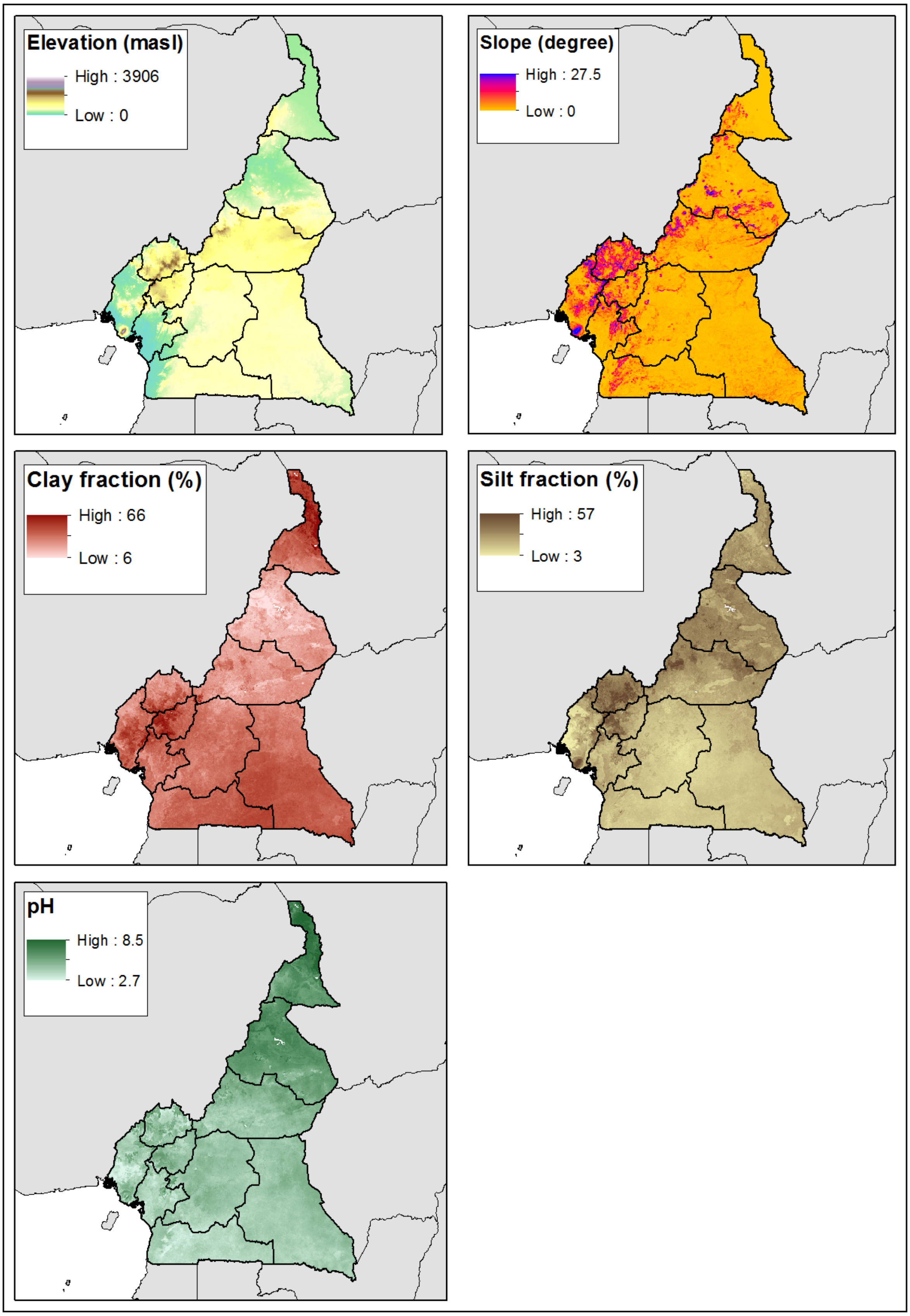

Figure 2S. The results of the Monte Carlo validation procedure. The solid line is the observed variogram and the shaded area corresponds to the 95% bandwidth. The results lead us to conclude that the data are compatible with the assumption of an exponential spatial correlation function.

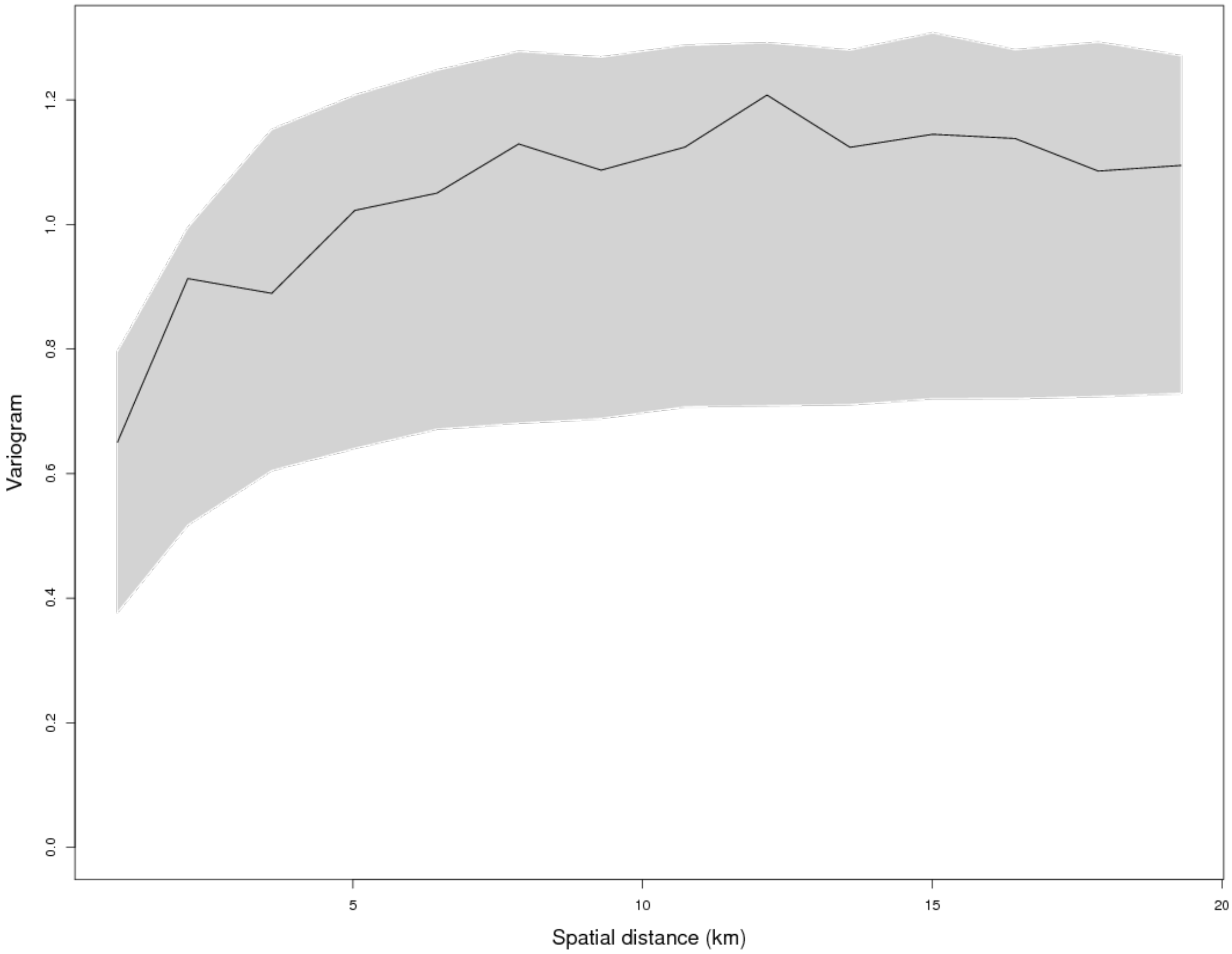

Figure 3S Partial dependence plots of the relative contribution of climate, vegetation and water related covariates to the boosted regression tree (BRT) model for podoconiosis, averaged over 100 ensembles. Blue lines represent the mean partial dependence over all 100 BRT ensembles and grey envelopes the standard deviation from the mean. The *y*-axis is the transformed logit response and *x*-axis is the full range of covariates values.

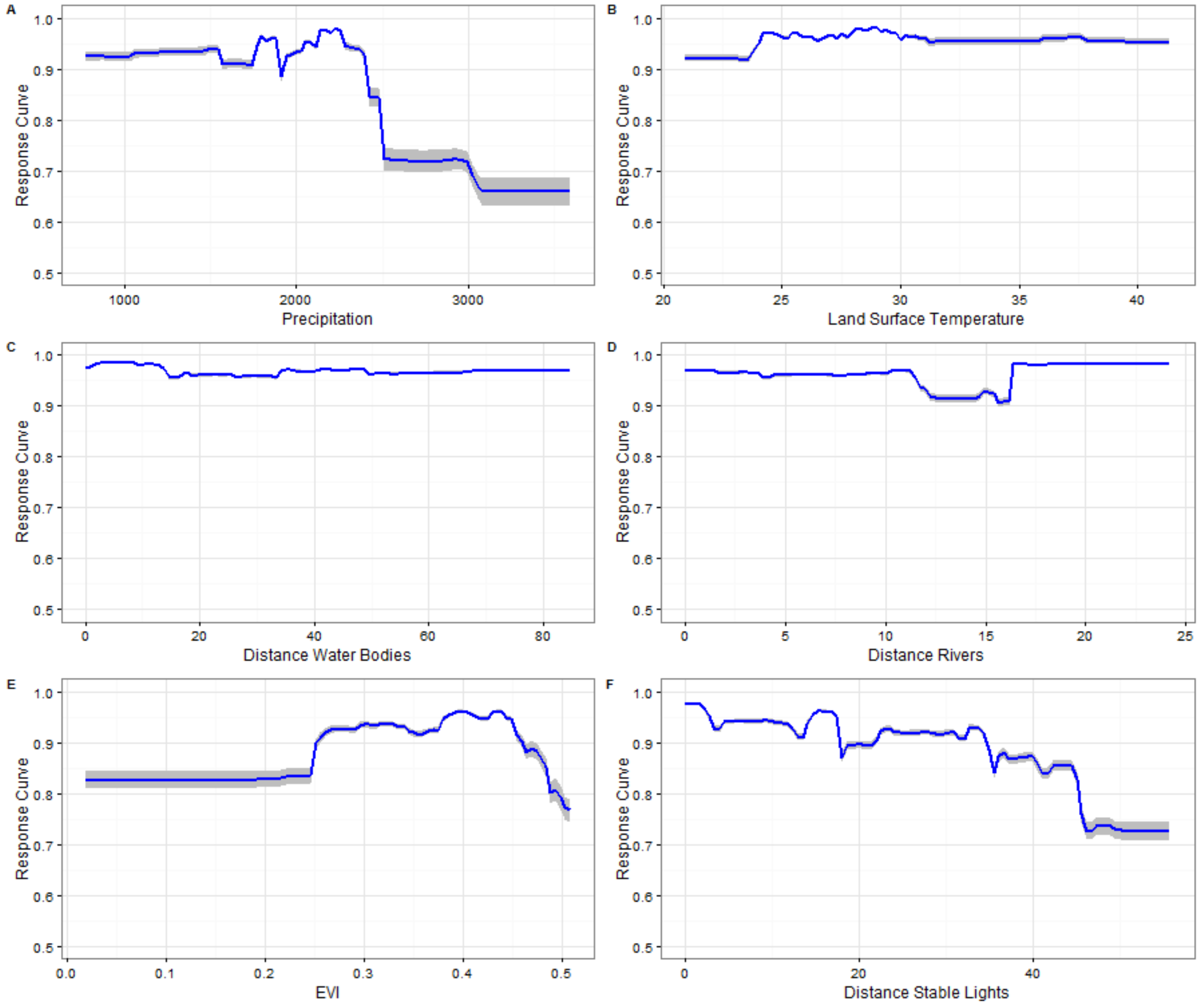

Figure 4S Partial dependence plots of the relative contribution of topography and soil composition related covariates to the boosted regression tree (BRT) model for podoconiosis, averaged over 100 ensembles. Blue lines represent the mean partial dependence over all 100 BRT ensembles and grey envelopes the standard deviation from the mean. The *y*-axis is the transformed logit response and *x*-axis is the full range of covariates values.

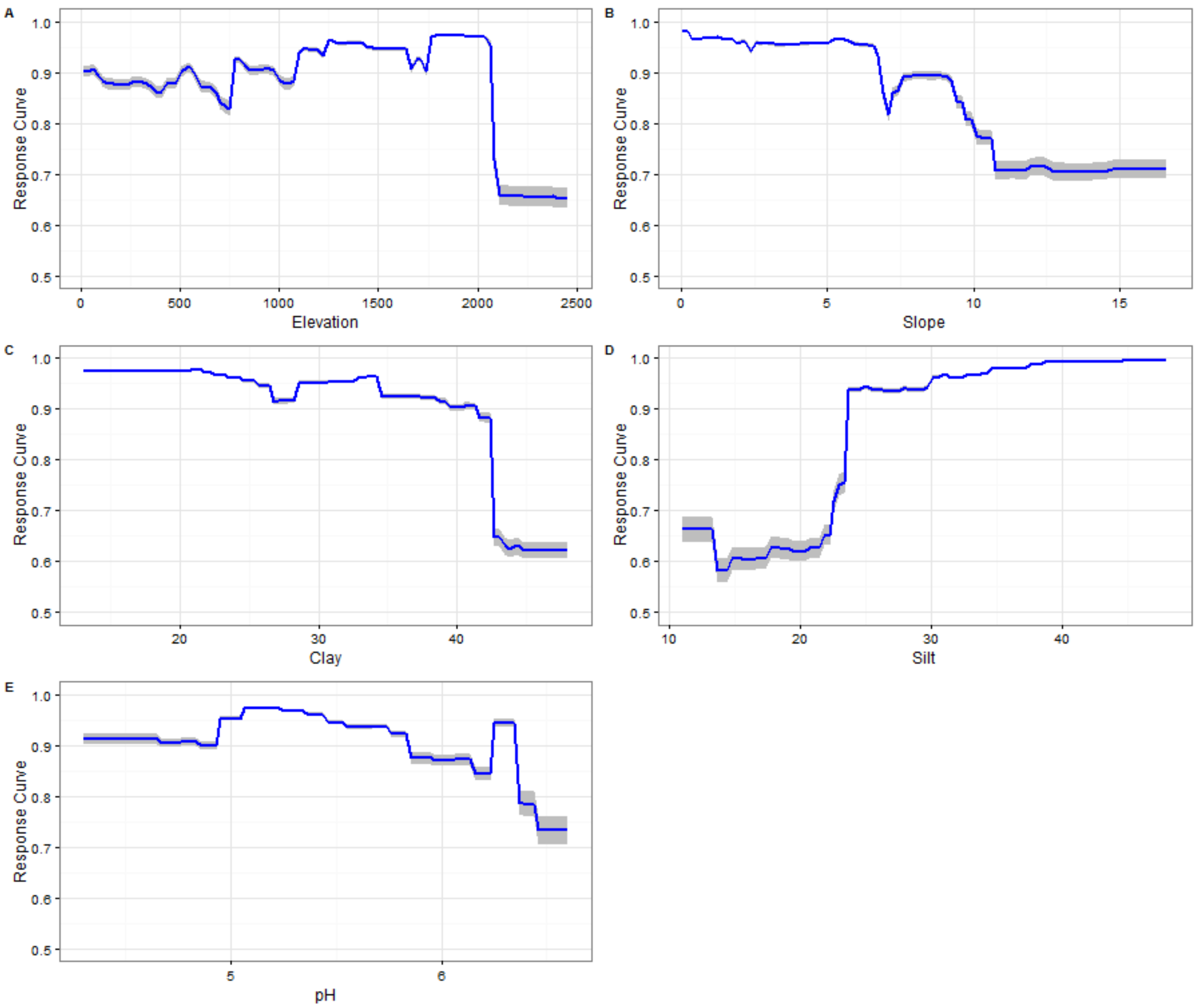

Figure 5S Partial dependence plots of the relative contribution of climate, vegetation and water related covariates to the random forest (RF) model for podoconiosis, averaged over 100 ensembles. Blue lines represent the mean partial dependence over all 100 RF ensembles and grey envelopes the standard deviation from the mean. The *y*-axis is the transformed logit response and *x*-axis is the full range of covariates values.

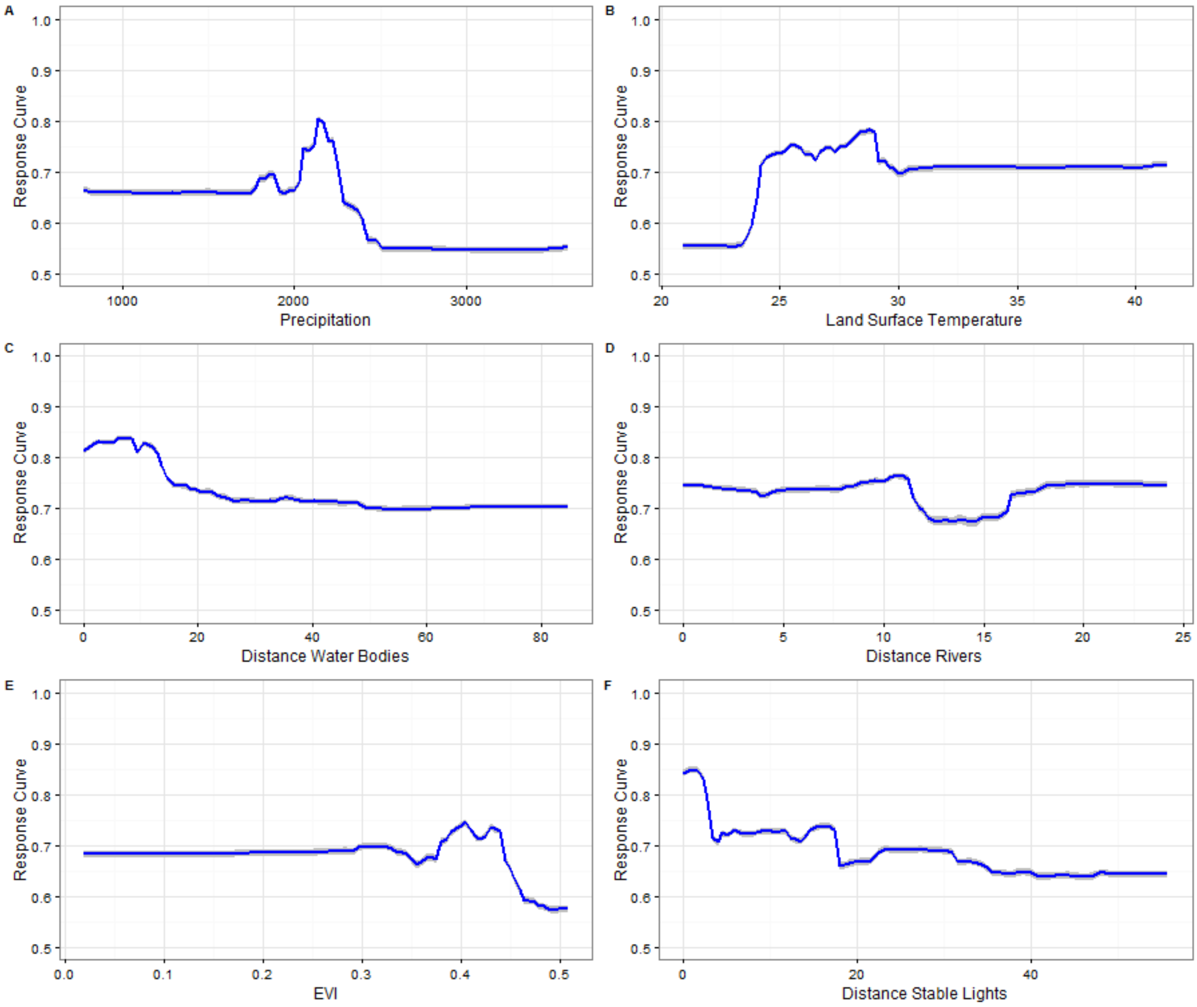

Figure 6S Partial dependence plots of the relative contribution of topography and soil composition related covariates to the random forest (RF) model for podoconiosis, averaged over 100 ensembles. Blue lines represent the mean partial dependence over all 100 BRT ensembles and grey envelopes the standard deviation from the mean. The *y*-axis is the transformed logit response and *x*-axis is the full range of covariates values.

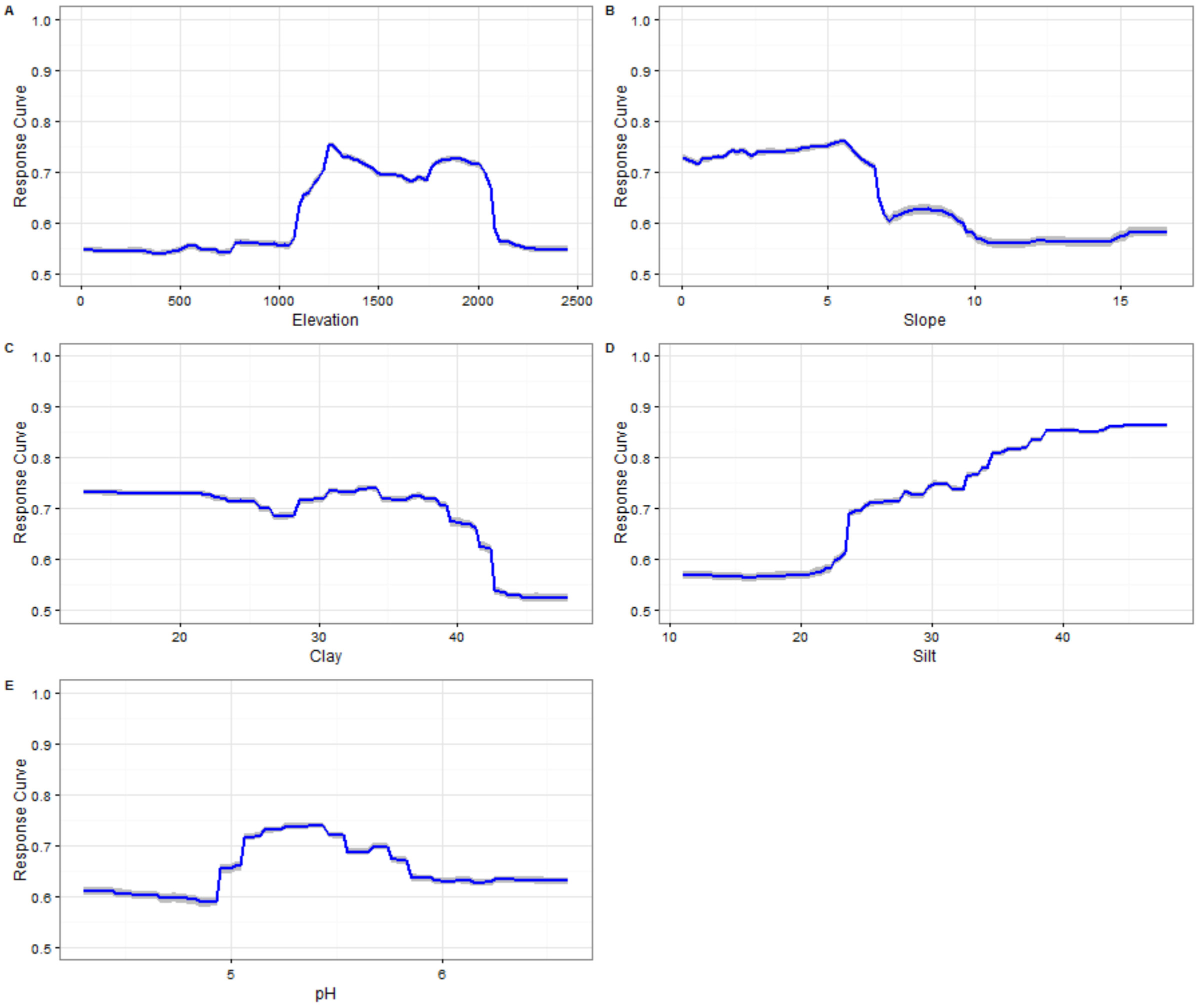

Figure 7S. Variable contribution of final ensemble models based on *boosted regression trees* and *random forest*. Variable contribution is provided as percentage, and it shows the relative contribution of selected environmental predictors to the final ensemble model of predicted podoconiosis occurrence.

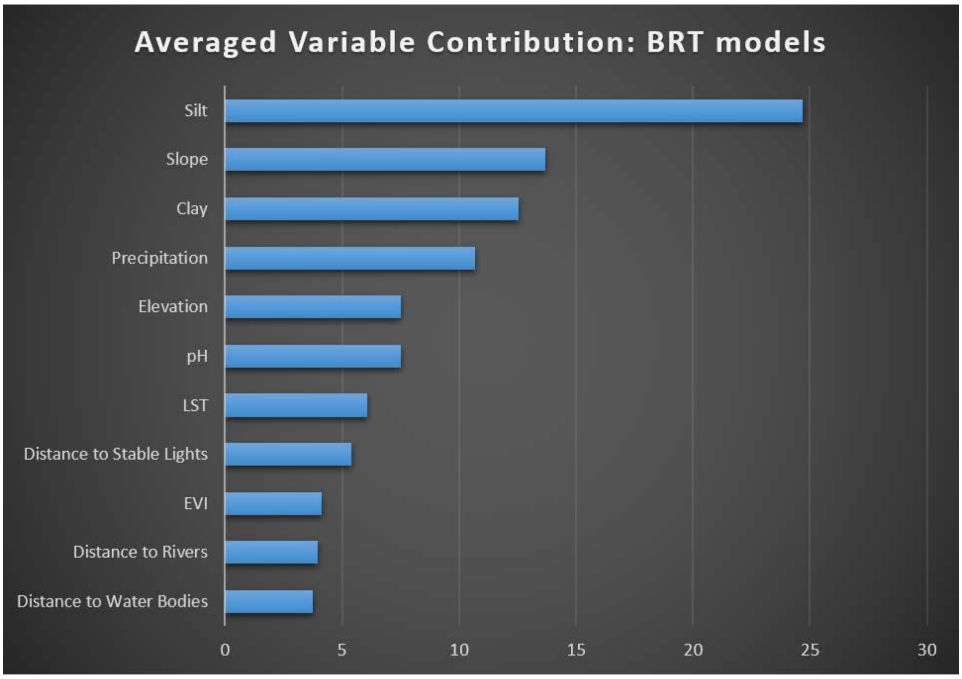

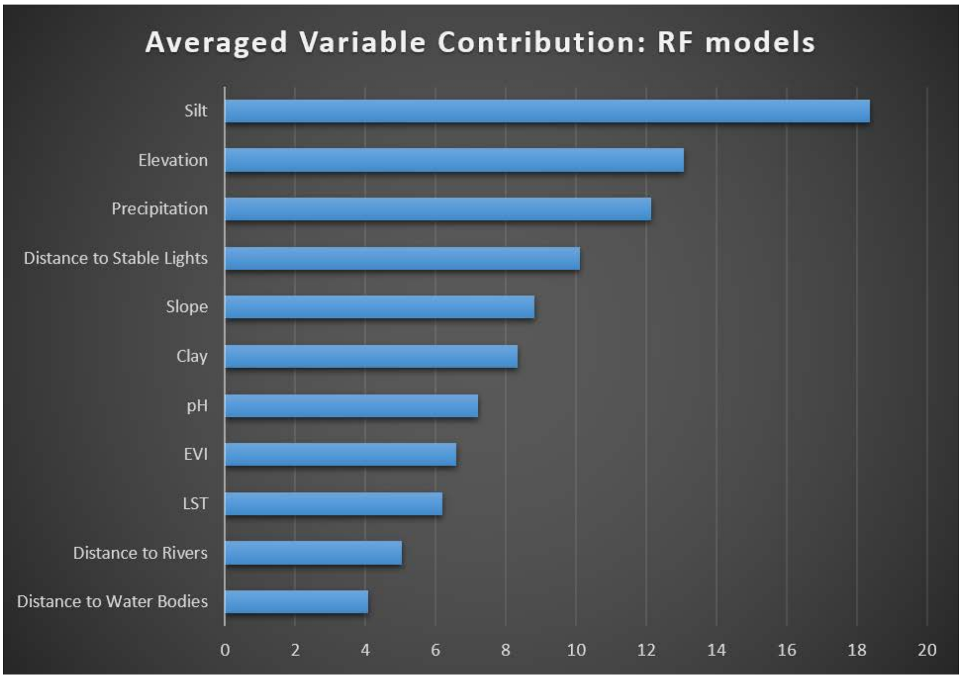

Predicted distribution and burden of podoconiosis in Cameroon.

Figure 8S Predicted occurrence of podoconiosis (A) and uncertainty range (B & C) across Cameroon. Optimal threshold was fitted to get better trade-off between sensitivity, specificity and proportion correctly classified (PCC).

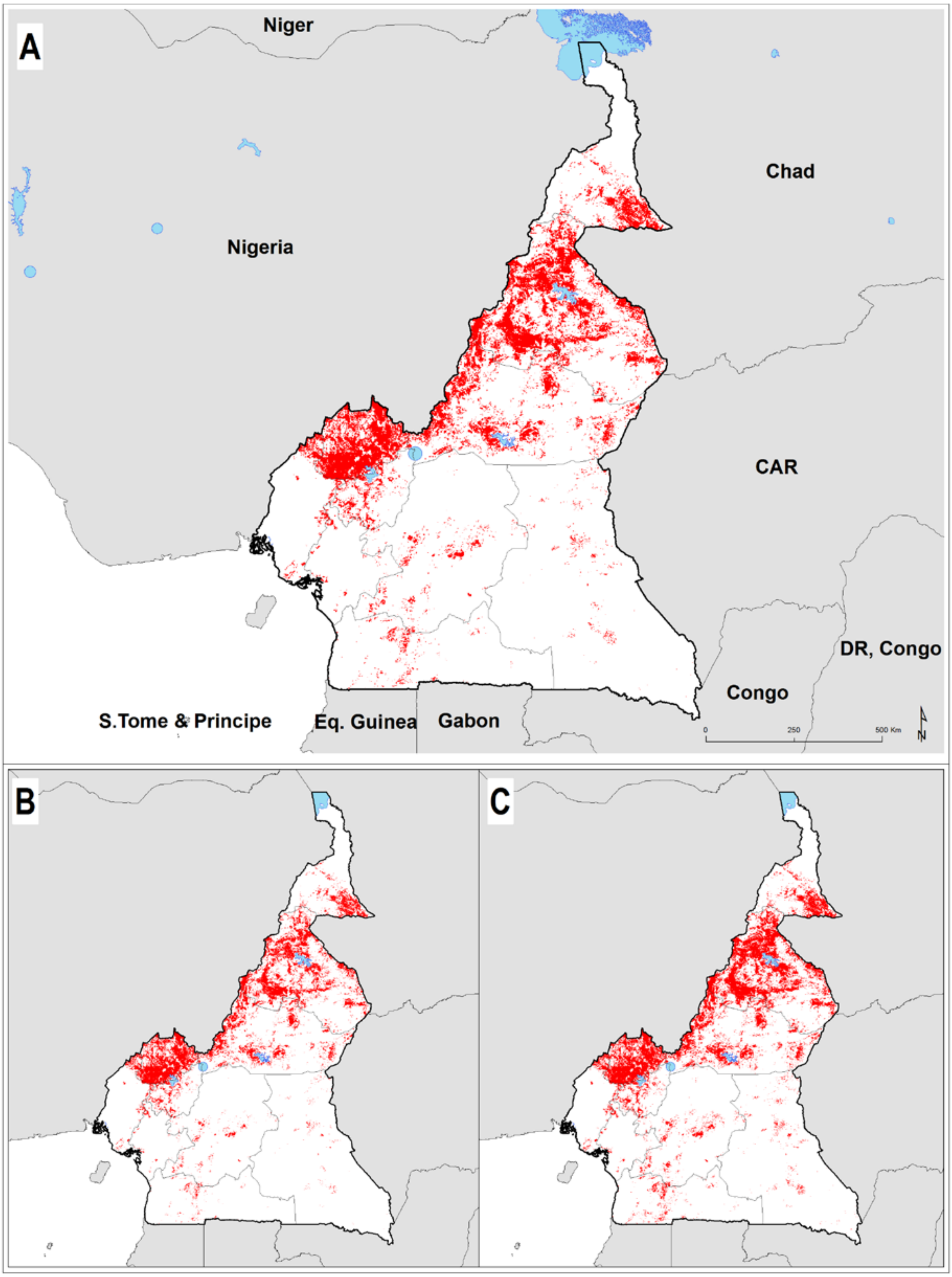

Predicted distribution and burden of podoconiosis in Cameroon.

Table 1S. Estimation of podoconiosis cases by Health District in Cameroon

SN	Country	Region	Health District	Podoconiosis Cases	Lower Bound	Upper Bound
1	Cameroon	Littoral	Ndom	3	0	18
2	Cameroon	Littoral	Ngambe	10	0	63
3	Cameroon	Littoral	Yabassi	0	0	1
4	Cameroon	Littoral	Nkongsamba	0	0	0
5	Cameroon	Littoral	Manjo	776	37	4054
6	Cameroon	Littoral	Melong	15	0	91
7	Cameroon	Littoral	Loum	0	0	2
8	Cameroon	Littoral	Abo	44	1	266
9	Cameroon	Littoral	Mbanga	176	4	1055
10	Cameroon	Littoral	Deido	680	15	4050
11	Cameroon	Littoral	Bonassama	149	3	888
12	Cameroon	Littoral	Mbangue	1	0	6
13	Cameroon	Littoral	Logbaba	3304	154	17573
14	Cameroon	Littoral	New Bell	9	0	53
15	Cameroon	Littoral	Nylon	0	0	0
16	Cameroon	Littoral	Pouma	771	17	4671
17	Cameroon	Littoral	Edea	10	0	60
18	Cameroon	Littoral	Nkondjock	8	0	46
19	Cameroon	Littoral	Manoka	0	0	1
20	Cameroon	Littoral	Boko	0	0	0
21	Cameroon	Littoral	Njombe Penja	259	6	1524
22	Cameroon	Littoral	Japoma	4	0	22
23	Cameroon	Littoral	Dibombari	0	0	0
24	Cameroon	Littoral	Cite Palmiers	0	0	0
25	Cameroon	Adamaoua	Tignere	94	2	564
26	Cameroon	Adamaoua	Ngaoundal	240	5	1440
27	Cameroon	Adamaoua	Meiganga	464	10	2770
28	Cameroon	Adamaoua	Bankim	104	2	621
29	Cameroon	Adamaoua	Banyo	215	5	1290
30	Cameroon	Adamaoua	Djohong	109	2	656
31	Cameroon	Adamaoua	Tibati	70	1	421
32	Cameroon	Adamaoua	Ngaoundere Rural	29	1	174
33	Cameroon	Adamaoua	Ngaoundere Urbain	956	20	5758
34	Cameroon	Central	Djoungolo	238	5	1447
35	Cameroon	Central	Mbalmayo	76	2	456
36	Cameroon	Central	Yoko	1	0	3
37	Cameroon	Central	Efoulan	730	17	4268
38	Cameroon	Central	Mbankomo	104	3	595
39	Cameroon	Central	Evodoula	6	0	35
40	Cameroon	Central	Mfou	0	0	0

Predicted distribution and burden of podoconiosis in Cameroon.

SN	Country	Region	Health District	Podoconiosis Cases	Lower Bound	Upper Bound
41	Cameroon	Central	Obala	15	0	89
42	Cameroon	Central	Ebebda	43	1	266
43	Cameroon	Central	Bafia	389	8	2344
44	Cameroon	Central	Ntui	17	0	101
45	Cameroon	Central	Elig Mfomo	19	0	115
46	Cameroon	Central	Mbandjock	224	5	1361
47	Cameroon	Central	Nanga Eboko	34	1	205
48	Cameroon	Central	Akonolinga	69	1	435
49	Cameroon	Central	Ngog Mapubi	80	2	483
50	Cameroon	Central	Eseka	142	3	847
51	Cameroon	Central	Awae	226	6	1265
52	Cameroon	Central	Esse	12	0	73
53	Cameroon	Central	Soa	73	2	452
54	Cameroon	Central	Monatele	7	0	41
55	Cameroon	Central	Ngoumou	153	3	902
56	Cameroon	Central	Ayos	0	0	1
57	Cameroon	Central	Ndikinimeki	56	1	331
58	Cameroon	Central	Cite Verte	4	0	24
59	Cameroon	Central	Nkolbissong	1	0	5
60	Cameroon	Central	Biyem Assi	598	14	3614
61	Cameroon	Central	Saa	19	0	116
62	Cameroon	Central	Nkolndongo	3936	99	23065
63	Cameroon	Central	Okola	0	0	0
64	Cameroon	East	Abong Mbang	16	0	94
65	Cameroon	East	Messamena	0	0	0
66	Cameroon	East	Garoua Boulai	13	0	78
67	Cameroon	East	Lomie	11	0	66
68	Cameroon	East	Moloundou	5	0	30
69	Cameroon	East	Yokadouma	42	1	254
70	Cameroon	East	Ndelele	29	1	177
71	Cameroon	East	Mbang	5	0	30
72	Cameroon	East	Batouri	193	6	1070
73	Cameroon	East	Betare Oya	6	0	36
74	Cameroon	East	Kette	18	0	106
75	Cameroon	East	Nguelemendouka	0	0	0
76	Cameroon	East	Bertoua	524	11	3135
77	Cameroon	East	Doume	54	1	318
78	Cameroon	Extreme North	Kolofata	143	3	870
79	Cameroon	Extreme North	Meri	31	1	186
80	Cameroon	Extreme North	Bogo	195	4	1173
81	Cameroon	Extreme North	Maroua 2	286	6	1786
82	Cameroon	Extreme North	Tokombere	56	1	333

Predicted distribution and burden of podoconiosis in Cameroon.

SN	Country	Region	Health District	Podoconiosis Cases	Lower Bound	Upper Bound
83	Cameroon	Extreme North	Gazawa	29	1	179
84	Cameroon	Extreme North	Kaele	231	5	1392
85	Cameroon	Extreme North	Guidiguis	213	5	1254
86	Cameroon	Extreme North	Guere	244	5	1468
87	Cameroon	Extreme North	Kar Hay	419	9	2520
88	Cameroon	Extreme North	Bourha	70	2	418
89	Cameroon	Extreme North	Moutourwa	74	2	440
90	Cameroon	Extreme North	Pette	105	2	625
91	Cameroon	Extreme North	Goulfey	0	0	0
92	Cameroon	Extreme North	Mogode	35	1	212
93	Cameroon	Extreme North	Hina	280	6	1696
94	Cameroon	Extreme North	Maroua 3	496	10	3014
95	Cameroon	Extreme North	Koza	24	1	142
96	Cameroon	Extreme North	Mokolo	14	0	85
97	Cameroon	Extreme North	Kousseri	28	1	173
98	Cameroon	Extreme North	Maroua 1	35	1	212
99	Cameroon	Extreme North	Makary	0	0	0
100	Cameroon	Extreme North	Mada	6	0	35
101	Cameroon	Extreme North	Roua	184	4	1111
102	Cameroon	Extreme North	Yagoua	540	12	3212
103	Cameroon	Extreme North	Vele	424	9	2520
104	Cameroon	Extreme North	Moulvoudaye	239	5	1439
105	Cameroon	Extreme North	Mindif	179	4	1075
106	Cameroon	Extreme North	Mora	289	6	1738
107	Cameroon	Extreme North	Maga	315	7	1901
108	Cameroon	North	Tchollire	210	5	1260
109	Cameroon	North	Poli	353	8	2116
110	Cameroon	North	Rey Bouba	232	5	1387
111	Cameroon	North	Pitoa	219	5	1302
112	Cameroon	North	Bibemi	130	3	786
113	Cameroon	North	Ngong	72	2	434
114	Cameroon	North	Garoua Ii	2405	52	14612
115	Cameroon	North	Lagdo	44	1	268
116	Cameroon	North	Garoua I	203	4	1231
117	Cameroon	North	Gaschiga	135	3	795
118	Cameroon	North	Figuil	209	5	1249
119	Cameroon	North	Touboro	297	8	1749
120	Cameroon	North	Golombe	115	2	694
121	Cameroon	North	Mayo Oulo	255	5	1538
122	Cameroon	North	Guider	927	20	5526
123	Cameroon	West	Batcham	59	1	350
124	Cameroon	West	Penka Michel	142	3	856

Predicted distribution and burden of podoconiosis in Cameroon.

SN	Country	Region	Health District	Podoconiosis Cases	Lower Bound	Upper Bound
125	Cameroon	West	Bandjoun	121	3	729
126	Cameroon	West	Mbouda	291	6	1732
127	Cameroon	West	Dschang	158	3	942
128	Cameroon	West	Bangourain	51	1	309
129	Cameroon	West	Bangangte	376	8	2241
130	Cameroon	West	Bamendjou	392	11	2205
131	Cameroon	West	Baham	117	3	701
132	Cameroon	West	Bandja	74	2	448
133	Cameroon	West	Foumban	124	3	753
134	Cameroon	West	Malentouen	50	1	296
135	Cameroon	West	Foumbot	127	3	768
136	Cameroon	West	Santchou	86	2	514
137	Cameroon	West	Kekem	181	4	1095
138	Cameroon	West	Kouoptamo	3	0	18
139	Cameroon	West	Mifi	1117	24	6749
140	Cameroon	West	Massangam	44	1	262
141	Cameroon	West	Galim	170	4	1028
142	Cameroon	West	Bafang	230	5	1370
143	Cameroon	South	Ebolowa	573	13	3419
144	Cameroon	South	Meyomessala	18	0	104
145	Cameroon	South	Kribi	196	4	1197
146	Cameroon	South	Lolodorf	14	0	84
147	Cameroon	South	Ambam	26	1	155
148	Cameroon	South	Zoetele	4	0	23
149	Cameroon	South	Sangmelima	7	0	42
150	Cameroon	South	Olamze	17	0	103
151	Cameroon	South	Djoum	2	0	15
152	Cameroon	South	Mvangan	18	0	110
153	Cameroon	South West	Fontem	601	13	3586
154	Cameroon	South West	Kumba	322	7	1842
155	Cameroon	South West	Akwaya	99	2	586
156	Cameroon	South West	Ekondo Titi	0	0	0
157	Cameroon	South West	Buea	89	3	506
158	Cameroon	South West	Mbonge	30	1	179
159	Cameroon	South West	Bakassi	1	0	9
160	Cameroon	South West	Eyumodjock	0	0	0
161	Cameroon	South West	Wabane	193	4	1166
162	Cameroon	South West	Tiko	87	3	485
163	Cameroon	South West	Limbe	1108	25	6594
164	Cameroon	South West	Muyuka	10	0	59
165	Cameroon	South West	Konye	0	0	0
166	Cameroon	South West	Tombel	18	0	111

Predicted distribution and burden of podoconiosis in Cameroon.

SN	Country	Region	Health District	Podoconiosis Cases	Lower Bound	Upper Bound
167	Cameroon	South West	Nguti	17	0	103
168	Cameroon	South West	Mamfe	189	5	1110
169	Cameroon	South West	Bangem	44	1	257
170	Cameroon	South West	Mundemba	0	0	1
171	Cameroon	North West	Bafut	541	23	2852
172	Cameroon	North West	Fundong	131	5	711
173	Cameroon	North West	Nwa	204	6	1157
174	Cameroon	North West	Benakuma	222	5	1310
175	Cameroon	North West	Tubah	251	12	1287
176	Cameroon	North West	Ako	142	4	826
177	Cameroon	North West	Bamenda	627	54	2707
178	Cameroon	North West	Bali	409	18	2112
179	Cameroon	North West	Ndop	304	19	1577
180	Cameroon	North West	Wum	140	3	827
181	Cameroon	North West	Santa	226	6	1281
182	Cameroon	North West	Njikwa	145	3	854
183	Cameroon	North West	Mbengwi	479	24	2396
184	Cameroon	North West	Nkambe	618	18	3512
185	Cameroon	North West	Ndu	225	8	1237
186	Cameroon	North West	Oku	127	5	690
187	Cameroon	North West	Batibo	888	40	4538
188	Cameroon	North West	Kumbo East	349	11	1971
189	Cameroon	North West	Kumbo West	251	11	1307
**National Total**				**41,556**	**1,170**	**240,992**
